# Neural integration underlying naturalistic prediction flexibly adapts to varying sensory input rate

**DOI:** 10.1038/s41467-021-22632-z

**Published:** 2021-05-11

**Authors:** Thomas J. Baumgarten, Brian Maniscalco, Jennifer L. Lee, Matthew W. Flounders, Patrice Abry, Biyu J. He

**Affiliations:** 1 Neuroscience Institute, New York University School of Medicine, New York, NY USA; 2 Institute of Clinical Neuroscience and Medical Psychology, Medical Faculty, Heinrich Heine University Düsseldorf, Düsseldorf, Germany; 3 Neuroscience Graduate Program, New York University, New York, NY USA; 4 CNRS, Laboratoire de Physique, Université de Lyon, ENS Lyon, Lyon, France; 5 Departments of Neurology, Neuroscience and Physiology, and Radiology, New York University School of Medicine, New York, NY USA

**Keywords:** Perception, Sensory processing

## Abstract

Prediction of future sensory input based on past sensory information is essential for organisms to effectively adapt their behavior in dynamic environments. Humans successfully predict future stimuli in various natural settings. Yet, it remains elusive how the brain achieves effective prediction despite enormous variations in sensory input rate, which directly affect how fast sensory information can accumulate. We presented participants with acoustic sequences capturing temporal statistical regularities prevalent in nature and investigated neural mechanisms underlying predictive computation using MEG. By parametrically manipulating sequence presentation speed, we tested two hypotheses: neural prediction relies on integrating past sensory information over fixed time periods or fixed amounts of information. We demonstrate that across halved and doubled presentation speeds, predictive information in neural activity stems from integration over fixed amounts of information. Our findings reveal the neural mechanisms enabling humans to robustly predict dynamic stimuli in natural environments despite large sensory input rate variations.

## Abstract (web summary)

Predicting future sensory input based on past sensory information is essential for organisms to effectively adapt their behaviour in dynamic environments. The authors identify the neural mechanisms enabling humans to predict dynamic stimuli in natural environments despite large sensory input rate variations.

## Introduction

The sensory information an organism receives in the natural environment is highly structured spatiotemporally due to statistical regularities inherent to natural stimuli^1,2^. Such regularities give rise to informational redundancy^3^, which organisms can exploit to effectively predict future stimuli. This allows organisms to generate predictions about their environment and produce adaptive behaviors, providing key benefits for survival^4^. Unsurprisingly, prediction of future stimuli has been empirically shown in humans in multiple contexts, ranging from visual scene perception^5^ and gaze control^6^ to object recognition^7^ and auditory speech perception^8^. Clinically, deficits in sensory prediction are considered to be a fundamental impairment of information processing in multiple psychiatric disorders^9^.

Effective stimulus prediction relies on correctly extrapolating past sensory information. This requires the nervous system to integrate sensory information over time, a process that we refer to as sensory history integration (SHI). Specifically, organisms need to accumulate past sensory information to extract statistical regularities (e.g., a melody), as such regularities cannot be inferred from single stimuli (e.g., a tone) alone. However, SHI is more than mere accumulation, as it requires selection of prediction-relevant sensory information and continuous updating to account for any changes in sensory input (e.g., the start of a new song). SHI thus represents a core computation for sensory prediction, as it provides a dynamically updated representation of past sensory input, from which a limited subset of probable future stimuli can be derived.

A central challenge for SHI is to determine how much past information should be integrated to ensure reliable predictions. Time-varying natural stimuli contain long-range temporal correlations that are rich and complex^10^. Integrating insufficient amounts of information yields inaccurate estimation of stimulus statistics and hence poor prediction. However, integration cannot reach back into sensory history infinitely, as the amount of information represented in neural systems is limited by biological and computational resources^11^. Neural implementation of SHI must therefore find a compromise between the minimal integrated information necessary for a sufficiently precise prediction and an integration bottleneck due to biological constraints.

In addition, the rate with which the nervous system receives sensory information is crucial for SHI, as it determines how quickly sensory information can in principle be integrated. The rate of sensory information arrival varies greatly in natural environments, for example in bird songs^12^ and human speech^13^. A previous fMRI study showed that neural responses in linguistic and extra-linguistic brain areas can flexibly scale in time across a 2–3-fold change in the speed of speech stimuli^14^. However, it remains unknown how variations in the rate of sensory information arrival influence SHI supporting prediction of future sensory input. Here, we address this open question by testing two alternative hypotheses about SHI underlying predictive neural computation given varying rates of sensory information arrival.

**Fig. 1:**
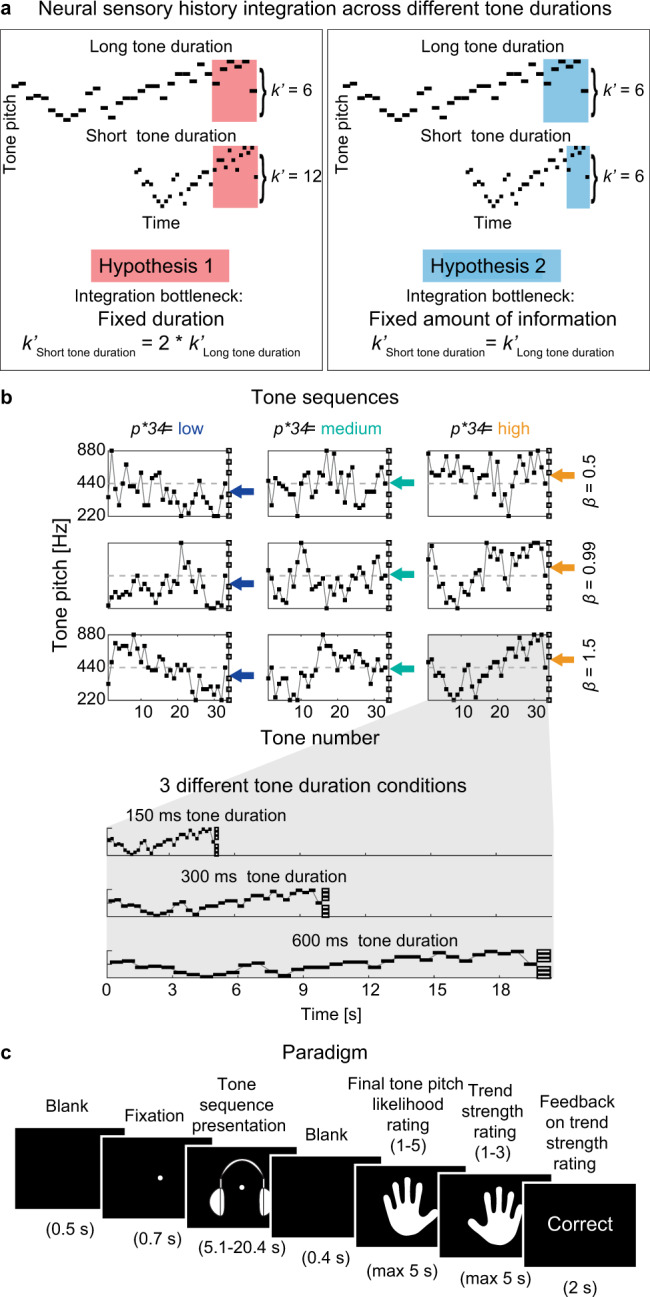
Hypotheses, stimuli, and paradigm. **a** Hypotheses. Neural sensory history integration (SHI) can be limited by a fixed duration (Hypothesis 1, highlighted red, temporal bottleneck) or a fixed amount of information (Hypothesis 2, highlighted blue, informational bottleneck), resulting in a different number of neurally integrated tones ($k\mbox{'}$) across-tone duration conditions. **b** Full stimulus set. Tone sequences consisted of 34 tones [black squares] ordered by temporal dependence level ($\beta$, rows) and theoretically predicted final tone (${p}_{34}^{\ast }$ [color-coded arrows], columns). For each beta level, we generated three sequences with tone pitch between 220 and 880 Hz. Sequences were chosen to have a ${p}_{34}^{\ast }$ lower than 440 Hz (column 1, blue arrows), equal to 440 Hz (column 2, turquoise arrows), or higher than 440 Hz (column 3, yellow arrows). For all sequences, the penultimate tone (${p}_{33}$) was 440 Hz. The final presented tone (${p}_{34}$ [empty black squares]) was pseudo-randomly drawn from one of six possible tone pitch values at 4, 8, or 12 semitones above or below 440 Hz. Sequences were presented with different tone durations (150 ms, 300 ms, 600 ms per tone). **c** Trial structure. After stimulus presentation, subjects rated the final tone pitch likelihood given the previous sequence information on a scale of 1–5. Subsequently, subjects rated the trend strength (i.e., beta level) of the presented sequence on a scale of 1–3.

First, SHI may operate over fixed time windows. According to this hypothesis, the amount of neurally integrated sensory information inversely scales with stimulus presentation rate (Fig. 1a, Hypothesis 1: Temporal bottleneck). This hypothesis is supported by evidence showing that sensory processing has an intrinsically stable (i.e., input-independent) temporal regime. Mechanistically, fixed temporal limitations in neural information processing can result from low-level biophysical constraints^15,16^. At the population level, prevalent neural oscillations in sensory cortices operate at intrinsically stable frequencies, which are tightly linked to stimulus processing^17,18^ and perception^19,20^.

Alternatively, SHI may operate over fixed amounts of information, which requires integration windows to scale flexibly in time to adapt to different stimulus presentation rates (Fig. 1a, Hypothesis 2: Informational bottleneck). This hypothesis is supported by behavioral studies in humans showing robust perceptual performance across varying stimulus presentation rates^21,22^. Neural evidence also suggests the plausibility of flexible SHI: e.g., at the single-cell level, neurons in the primary auditory cortex process acoustic features at multiple timescales^23^; at the population level, fMRI responses flexibly scale to stimulus presentation speed^14^. Computationally, recurrent neural networks can recognize identical stimuli presented at different speeds^24^.

At present, the relevance of these findings to predictive neural computation remains unclear. To adjudicate between the temporal bottleneck and informational bottleneck hypotheses regarding neural SHI underlying predictions about future sensory input, we presented human subjects with acoustic sequences exhibiting precisely manipulated predictive information and parametrically varied presentation speed. Importantly, we constructed these stimuli to capture the temporal regularities pervasive in the natural environment in order to investigate predictive computation based on natural temporal regularities^10,25^. We present findings demonstrating that neural predictions integrate a constant number of stimuli across a four-fold change in stimulus presentation rates and are thus constrained by an informational bottleneck.

## Results

### Paradigm and behavior

We presented subjects with auditory tone sequences exhibiting statistical regularities similar to natural acoustic stimuli (e.g., natural soundscapes, speech, and music). Within each tone sequence, pitch fluctuated over time in a temporally autocorrelated manner, such that the pitch of a given tone was statistically dependent on the pitch of preceding tones within that sequence. Specifically, fluctuations adhered to a temporal power spectrum characterized by P ∝ 1 / f ^β^, where 0 < *β* < 2, thereby mimicking the temporal statistical regularities commonly seen in dynamical natural stimuli^1,26,27^. In previous work, we have developed a mathematical framework to precisely manipulate predictive information in such sequences with naturalistic temporal regularities^25^.

In each trial, we presented subjects with an auditory tone sequence consisting of 34 concatenated, nonoverlapping pure tones without interstimulus interval (Fig. 1b; see Methods). Tone duration was kept constant within a sequence but systematically varied across trials (Fig. 1b, bottom), producing three conditions differing in sequence presentation speed: fast (150 ms/tone), medium (300 ms/tone), slow (600 ms/tone). Pitch fluctuations within each sequence exhibited one of three temporal dependence levels (quantified by the *β* parameter in the temporal power spectrum^28^), ranging from weak (*β* = 0.5) to medium (*β* = 0.99) to strong (*β* = 1.5). Importantly, all sequences converged on the same pitch (440 Hz) for the penultimate (33rd) tone, at which timepoint each sequence’s unique history predicted a specific upcoming tone pitch (${p}_{34}^{\ast }$). This crucial design provided a time window wherein sensory input was constant across trials, yet sequence history and the predicted upcoming sensory input differed across trials, allowing us to separate instantaneous sensory processing from predictive processing building up over the course of the sequence.

For each sequence, the theoretically predicted final tone pitch (${p}_{34}^{\ast }$) was derived from all preceding tone pitches in the sequence^10,25^. ${p}_{34}^{\ast }$ was not actually presented to the subject. Instead, the pitch of the actually presented 34th tone (${p}_{34}$) was pseudo-randomly drawn from six possible values located four, eight, or twelve semitones below/above 440 Hz. Consequently, for a listener who can extract predictive information provided by the temporal dependencies between tone pitches within a sequence, their judgment of final tone pitch likelihood—capturing their perception of how well the last tone fits into the preceding tone sequence—should be a function of both the presented final tone pitch (${p}_{34}$) and the theoretically predicted final tone pitch (${p}_{34}^{\ast }$). By rating final tone pitch likelihood on each trial (Fig. 1c), subjects therefore provided an indirect index of their prediction of the final tone pitch.

**Fig. 2:**
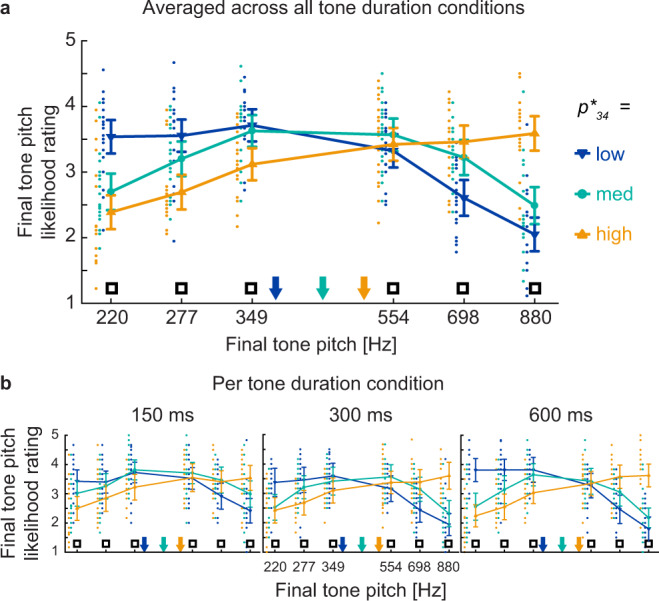
Subjects’ behavior demonstrates effective final tone pitch prediction (*n* = 20 participants). **a** Final tone pitch likelihood ratings averaged across all tone duration conditions. Final tone pitch likelihood rating (*y*-axis; 1 = unlikely, 5 = likely) is plotted as a function of presented final tone pitch (${p}_{34}$ [empty black squares], *x*-axis) and theoretically predicted final tone pitch (${p}_{34}^{\ast }$; color-coded). ${p}_{34}^{\ast }$ tone pitch is indicated on the *x*-axis by color-coded arrows. A repeated-measures ANOVA shows a significant interaction between ${p}_{34}$and ${p}_{34}^{\ast }$ [$F$_2,46_ = 36.96, $p$ < 0.001, ${{\rm{\eta }}}_{p}^{2}$ = 0.66], indicated by the crossover of the blue and yellow lines. Dots represent individual participant data. Data are presented as mean ± SEM across participants. **b** Final tone pitch likelihood ratings per tone duration condition. Same format as **a**.

To investigate the behavioral effect of final tone pitch prediction, subjects’ final tone pitch likelihood ratings were submitted to a three-way repeated-measures ANOVA (factors: 3 (tone duration) $\times$ 3 (${p}_{34}^{\ast }$) $\times$ 6 (${p}_{34}$); all *n* = 20; see Methods). There was a significant main effect of ${p}_{34}$ [$F$_2,41_ = 16.22, $p$ < 0.001, ${{\rm{\eta }}}_{p}^{2}$ = 0.46], accounting for the inverse U-shaped function where extreme ${p}_{34}$-values were rated as less likely (Fig. 2a). Importantly, a significant interaction was found between ${p}_{34}^{\ast }$ and ${p}_{34}$ [$F$_2,46_ = 36.96, $p$ < 0.001, ${{\rm{\eta }}}_{p}^{2}$ = 0.66], suggesting that subjects’ likelihood rating of the final tone depended on its predicted value given the previous sequence history. Across-tone duration conditions, when ${p}_{34}$ was high (880 Hz), participants rated it as more likely when ${p}_{34}^{\ast }$ was also high (paired $t$-test for low ${p}_{34}^{\ast }$ vs high ${p}_{34}^{\ast }$, $t$_19_ = −8.66, $p$ < 0.01, $d$ = −1.94). Likewise, when ${p}_{34}$ was low (220 Hz), participants rated it as more likely when ${p}_{34}^{\ast }$ was also low (low ${p}_{34}^{\ast }$ vs. high ${p}_{34}^{\ast }$, $t$_19_ = 5.12, $p$ < 0.01, $d$ = 1.15).

A significant interaction effect between ${p}_{34}^{\ast }$ and ${p}_{34}$ on final tone pitch likelihood ratings was further found within each tone duration condition using a two-way repeated-measures ANOVA (factors: 3 (${p}_{34}^{\ast }$) $\times$ 6 (${p}_{34}$); 150 ms: [$F$_5,97_ = 11.94, $p$ = 0.001, ${{\rm{\eta }}}_{p}^{2}$ = 0.39]; 300 ms: [$F$_4,79_ = 20.6, $p$ < 0.001, ${{\rm{\eta }}}_{p}^{2}$ = 0.52]; 600 ms: [$F$_4,71_ = 25.09, $p$ < 0.001, ${{\rm{\eta }}}_{p}^{2}$ = 0.57], Fig. 2b), demonstrating that subjects successfully predicted final tone pitch across a four-fold variation in stimulus presentation rate. Significant ${p}_{34}$ main effects and ${p}_{34}^{\ast }\times {p}_{34}$ interactions effects could further be replicated for both the first and the second half of the experiment (see Supplementary Notes for details), suggesting that behavioral performance is stable across the course of the experiment.

### Correlates of prediction and sensory history integration in slow arrhythmic neural activity

**Fig. 3:**
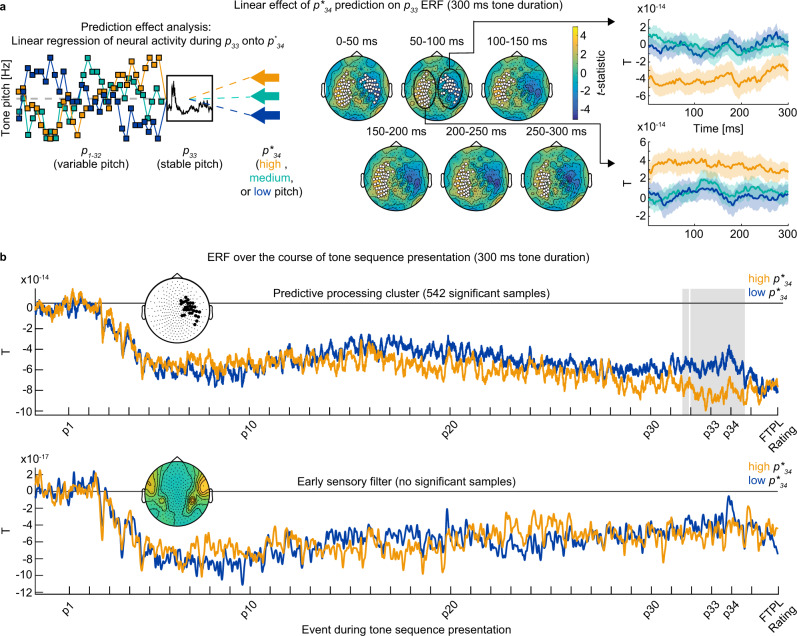
Slow arrhythmic neuromagnetic activity contains predictive information about upcoming tone pitch (*n* = 20 participants). **a** Prediction analysis schematic and group-level neuromagnetic correlates of theoretically predicted final tone pitch (${p}_{34}^{\ast }$ [color-coded]) prediction for data from the 300 ms tone duration condition. Non-baseline-corrected neuromagnetic activity averaged across 50 ms time windows during the penultimate tone (${p}_{33}$) was regressed onto ${p}_{34}^{\ast }$ to reveal sensor clusters where neuromagnetic activity is predictive of future tone pitch. Topoplots show $t$-values corresponding to a group-level one-sample $t$-test on regression coefficients for each sensor and time window. White dots indicate significant predictive processing clusters (all $p$ < 0.05, cluster-based permutation test, two-tailed). Right inset shows neuromagnetic activity (unit: T = Tesla) during ${p}_{33}$-presentation as a function of ${p}_{34}^{\ast }$, averaged across sensors for the left (bottom) and right (top) predictive processing cluster defined in the 50–100 ms time window. Data are presented as mean ± SEM across participants. **b** Event-related fields (ERF) over the course of tone sequence presentation (${p}_{1}$ = tone 1 within the current sequence) for tone sequences with low vs. high ${p}_{34}^{\ast }$ (example shown for data from the 300 ms tone duration condition). Top panel: ERF computed for the right predictive processing cluster defined from the 50–100 ms time window (inset and Fig. 3a). Bottom panel: ERF computed for the early sensory filter (inset shows corresponding sensor weights). Gray shading indicates significant differences between trials with low and high ${p}_{34}^{\ast }$ (all $p$ < 0.05, cluster-based permutation test, two-tailed). Data are presented as mean across participants.

To elucidate neural activity underlying predictive performance, we first investigated if neuromagnetic activity during the penultimate (33rd) tone (${p}_{33}$) contains information about the theoretically predicted final tone pitch, ${p}_{34}^{\ast }$ (left inset in Fig. 3a; see Methods). Since the pitch of ${p}_{33}$ was constant (440 Hz) across all trials, this analysis was able to identify neural activity underlying prediction without being affected by instantaneous sensory processing. Specifically, we computed a linear regression of neuromagnetic activity during ${p}_{33}$ (averaged across 50-ms-length sliding windows for each sensor) as a function of ${p}_{34}^{\ast }$. Analyzed neural activity was not baseline corrected, which allowed us to capture the continuous buildup of predictive information emerging over the course of the tone sequence^25,29^.

Group-level sensor clusters carrying significant predictive information about ${p}_{34}^{\ast }$ were identified for all tone duration conditions and are subsequently labeled as predictive processing clusters (all *n* = 20). The results of the medium (300 ms) tone duration condition (Fig. 3a) were selected to define sensors of interest used in further analyses. Two significant bilateral predictive processing clusters were identified in the first time window (0–50 ms: left cluster: 41 sensors, $p$ = 0.005, ${d}_{{{\mathrm{cluster}}}}$ = 5.2; right cluster: 27 sensors, $p$ = 0.024, ${d}_{{{\mathrm{cluster}}}}$ = 2.9, cluster-based permutation test, two-tailed; see Methods) and the second time window (50–100 ms: left cluster: 38 sensors, $p$ = 0.016, ${d}_{{{\mathrm{cluster}}}}$ = 3.8; right cluster: 29 sensors, $p$ = 0.024, ${d}_{{{\mathrm{cluster}}}}$ = 2.8). In the remaining time windows, the left predictive processing cluster remained significant (100–150 ms: 34 sensors, $p$ = 0.014, ${d}_{{{\mathrm{cluster}}}}$ = 3.8; 150–200 ms: 43 sensors, $p$ = 0.002, ${d}_{{{\mathrm{cluster}}}}$ = 5.3; 200–250 ms: 42 sensors, $p$ = 0.002, ${d}_{{{\mathrm{cluster}}}}$ = 6; 250–300 ms: 34 sensors, $p$ = 0.017, ${d}_{{{\mathrm{cluster}}}}$ = 3.3). Averaged across sensors within a cluster, neural activity during the penultimate tone (${p}_{33}$, always at 440 Hz) varied according to the predicted upcoming tone pitch (${p}_{34}^{\ast }$; right inset in Fig. 3a shows an illustrative example for the left and right cluster at 50–100 ms). Analysis of short (150 ms) and long (600 ms) tone duration conditions revealed a comparable topography (Supplementary Fig. 1), although only the right predictive processing cluster reached significance therein. In addition, the neural prediction effects were relatively stable across the first and second halves of the experiment (see Supplementary Notes and Supplementary Fig. 2).

We next investigated how the predictive information contained in neural activity builds up over the course of tone sequence presentation. To this end, we plotted time-resolved neural activity (i.e., broadband event-related fields) over the course of the entire sequence and contrasted it between tone sequences converging on low vs. high ${p}_{34}^{\ast }$ (see Methods). In predictive processing clusters, neural activity increasingly diverged between low vs. high ${p}_{34}^{\ast }$ trials, manifesting in continuous epochs—towards the end of the sequence—showing a significant difference in all tone duration conditions (see Fig. 3b, top for an example from the 300 ms condition; see Supplementary Fig. 3 for other conditions).

As a comparison, we also investigated neural activity projected through a spatial filter focusing on early sensory processing (subsequently labeled early sensory filter). Spatial filters were constructed based on sensor weights determined by the sensor-wise signal contribution during time windows covering the M100 evoked response to each tone (Fig. 3b, bottom). The M100 response represents a common auditory functional localizer^30^ known to focus on the initial stages of auditory stimulus processing in Heschl’s gyrus and primary auditory cortex^31^. Neural activity in this filter exhibited prominent event-related fields following the onset of each tone. However, no epochs showing a significant difference between low vs. high ${p}_{34}^{\ast }$ trials emerged in any tone duration condition (Fig. 3b, bottom; see Supplementary Fig. 3 for other conditions).

**Fig. 4:**
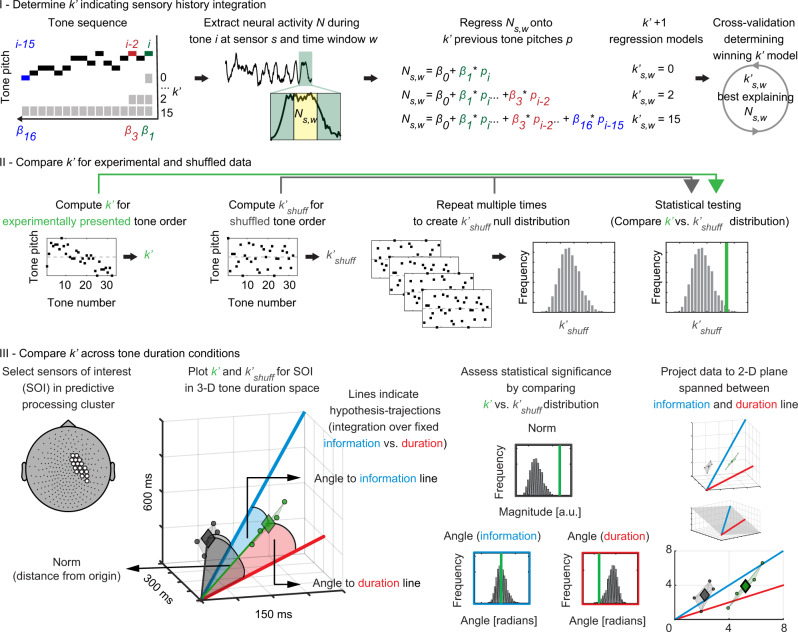
Sensory history integration analysis schematic. **I** Non-baseline-corrected MEG activity, ${N}_{s,w}$ for sensor $s$ and time window $w$, in response to the $i$-th tone (16≤ $i$ ≤32) is linearly regressed onto the current tone pitch (${p}_{i}$) and the pitch of $k\mbox{'}$ preceding tones. The $k\mbox{'}$-value providing the best model fit is determined by cross-validation, indicating the number of preceding tone pitches best explaining ${N}_{s,w}$. **II** $k\mbox{'}$-values from experimental data (green) are compared against a null distribution constructed by shuffling tone order within each sequence (${k}_{{shuff}}^{{\prime} }$, gray). **III** $k\mbox{'}$-values computed for predictive processing clusters are compared across-tone duration conditions within a 3-D coordinate system (axes represent tone duration-specific $k\mbox{'}$). Sensor-wise $k\mbox{'}$values are shown as green dots, ${k}_{{shuff}}^{{\prime} }$-values are shown in gray. Diamonds represent the center of mass for the respective distribution (for visualization only). Blue (informational bottleneck) and red (temporal bottleneck) orientation lines indicate the hypothesis-derived location of $k\mbox{'}$-values. Vector norm (i.e., distance from origin) and angle (i.e., angle to respective orientation lines) for $k\mbox{'}$-values are statistically compared against the shuffled null distribution (histograms). $k\mbox{'}$-values were additionally projected onto a 2-D plane defined by the orientation lines.

Since all predictive information must stem from integration of past sensory information, we next quantitatively assessed how SHI is embodied in neural activity. To this end, we determined how neuromagnetic activity at a given moment depends on the pitch of previously presented tones (analysis schematic in Fig. 4; see Methods). Non-baseline-corrected neuromagnetic activity after the onset of a given tone during the 2nd half of a sequence was extracted and averaged across 50-ms-length time windows. Subsequently, the activity at each sensor and time window was regressed onto the pitch of the current tone and ${k}^{{\prime} }$ previous tones, with ${k}^{{\prime} }$ ranging from 0 to 15 (corresponding to 1–16 integrated tones). A cross-validation approach was used to determine the ${k}^{{\prime} }$-value exhibiting the best model fit for the experimental data; these best-fit ${k}^{{\prime} }$-values indicate the number of previously presented tones whose pitch influences neural activity at the present moment. To statistically assess if SHI effects were significant, ${k}^{{\prime} }$-values from experimental data were compared against a null distribution generated by repeating the same analysis but with shuffled tone order within each sequence (${k}_{{{\mathrm{shuff}}}}^{{\prime} }$), corresponding to the null hypothesis that there is no systematic integration of previously presented tones. Widespread sensor clusters exhibiting significant SHI effects were identified for all tone duration conditions and across all time windows (see Supplementary Fig. 4). Next, we quantitatively assessed the dependence of neural SHI underlying prediction on stimulus presentation rate, and adjudicated between the informational bottleneck and temporal bottleneck hypotheses.

### Prediction relies on integrating a stable number of tones across stimulus presentation speeds

According to our two alternative hypotheses, SHI underlying prediction might operate over fixed time windows (Hypothesis 1: Temporal bottleneck) or fixed amounts of information (Hypothesis 2: Informational bottleneck). When the rate of stimulus presentation changes, these two hypotheses predict that the number of tones integrated by neural activity (estimated by ${k}^{{\prime} }$) either scales accordingly (Hypothesis 1) or remains the same (Hypothesis 2; Fig. 1a). Thus, Hypothesis 1 predicts a ${{k}^{{\prime} }}_{150{msTD}}=2{{k}^{{\prime} }}_{300{msTD}}=4{{k}^{{\prime} }}_{600{msTD}}$ relationship between ${k}^{{\prime} }$ across-tone duration conditions. Hypothesis 2, on the other hand, predicts ${{k}^{{\prime} }}_{150{msTD}}={{k}^{{\prime} }}_{300{msTD}}={{k}^{{\prime} }}_{600{msTD}}$. These two predictions can be visualized as two orientation lines in a three-dimensional space, where each dimension corresponds to ${k}^{{\prime} }$-values from a given tone duration condition (Fig. 4-III, red: duration line corresponding to Hypothesis 1; blue: information line corresponding to Hypothesis 2).

To test these hypotheses, we investigated where neural data lie in relation to these two hypothesis-derived orientation lines. To this end, we adopted two complementary approaches. First, we tested sensors within predictive processing clusters (i.e., sensor clusters showing a significant ${p}_{34}^{\ast }$ prediction effect) to focus on neural SHI underlying sensory prediction. Second, we made no a priori sensor selection and tested the two hypotheses across the entire sensor array (with cluster-based permutation test to correct for multiple comparisons) to check whether there is positive evidence for each hypothesis.

To focus on SHI underlying the ${p}_{34}^{\ast }$ prediction effect, comparison of ${k}^{{\prime} }$-values across different tone duration conditions was performed for predictive processing clusters defined from the 300 ms tone duration data (analysis schematic in Fig. 4; see Methods). For each sensor in the predictive processing cluster, its ${k}^{{\prime} }$-values from the three tone duration conditions computed for time windows corresponding to the respective predictive processing cluster were plotted as a point in the 3-D space (Fig. 4-III, left, green dots for illustration). To determine the overall magnitude of ${k}^{{\prime} }$-values (i.e., the length of SHI), we computed vector norm as the distance of each point to the origin. To determine the distance between neural data and hypothesis-derived orientation lines, we computed the angle between a vector connecting the origin and neural data and the respective orientation lines. Vector norm and angle were averaged across sensors and statistically evaluated by comparison against a null distribution generated from the norm and angle of shuffled data (i.e., ${k}_{{{\mathrm{shuff}}}}^{{\prime} }$-values from the three tone duration conditions; Fig. 4-III, left, gray dots and center histograms).

**Fig. 5:**
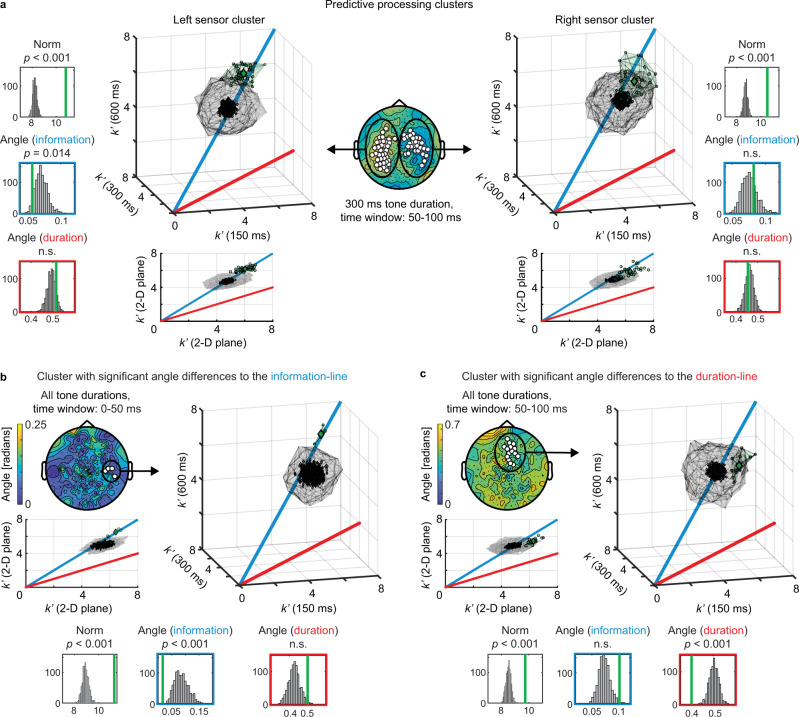
Sensory history integration operates differently across spatial locations, but predictive processing clusters exhibit flexible scaling to sensory input rate (*n* = 20 participants). **a** Across-tone duration-condition comparison of the number of preceding tones best explaining neuromagnetic activity ($k\mbox{'}$-values) from the left and right predictive processing cluster (300 ms tone duration condition, 50–100 ms time window). $k\mbox{'}$-values from experimental data in both sensor clusters (3-D and 2-D plots, green dots) reside significantly (top histograms, $p$ < 0.001, nonparametric permutation test, one-tailed) further away from origin at $k\mbox{'}$ ≈ 6 (i.e., 7 integrated tones) than shuffled data (gray dots). Experimental $k\mbox{'}$-values in the left sensor cluster reside significantly closer to the information line than shuffled data (left middle histogram, $p$ = 0.014, nonparametric permutation test, one-tailed). Additional results from predictive processing clusters defined using other time windows are shown in Supplementary Fig. 5. **b** A data-driven analysis across the entire sensor array identified a sensor cluster in which SHI behaves according to the informational bottleneck hypothesis. This sensor cluster overlaps with the right predictive processing cluster shown in **a**. Topoplot shows the angle between experimental data and the information line for all sensors. Significant sensors where the angle is smaller than shuffled data are shown in white ($p$ < 0.05, cluster-based permutation test, one-tailed). Here, $k\mbox{'}$-values reside significantly further away from origin (left histogram, $p$ < 0.001) and significantly closer to the information line than shuffled data (middle histogram, $p$ < 0.001). **c** A sensor cluster in which SHI behaves according to the temporal bottleneck hypothesis, identified by the data-driven analysis. This sensor cluster has minimal overlap with the predictive processing clusters. Topoplot shows the angle between experimental data and the duration line for all sensors, and significant sensors where the angle is smaller than shuffled data are shown in white ($p$ < 0.05, cluster-based permutation test, one-tailed). Here, $k\mbox{'}$-values reside significantly further away from origin (left histogram, $p$ < 0.001) and significantly closer to the duration line than shuffled data (right histogram, $p$ < 0.001).

Results for both sensor clusters during the 50–100 ms time window following tone onset are presented in Fig. 5a (see Supplementary Fig. 5 for the other time windows). Vector norm for ${k}^{{\prime} }$-values was found to be significantly larger than expected under the null distribution (mean norm: 10.67 ± 0.43 (SD), $p$ < 0.001; upper histograms in Fig. 5a and Supplementary Fig. 5; all *n* = 20), as can be seen from the real data lying further away from the origin than shuffled data (3-D plots). This suggests that there is significant sensory history integration in predictive processing clusters, as expected. Vector angle to the information line for ${k}^{{\prime} }$-values was significantly smaller than expected under the null distribution in the left sensor cluster (mean angle: 0.055 ± 0.033, $p$ = 0.014; left middle histogram in Fig. 5a; data from the 100–150 ms time window showed a trend effect: mean angle: 0.061 ± 0.027, $p$ = 0.065, Supplementary Fig. 5b). No significant effect was found for vector angle to the duration line in any of the predictive processing clusters. In a control analysis (Fig. 5, 2-D plots), we projected data onto a two-dimensional plane defined by the two hypothesis-derived orientation lines and recomputed vector norm and vector angle towards both orientation lines in this 2-D plane, which replicated all results from the 3-D analysis (Supplementary Table 1).

To investigate how SHI operates outside of sensors underlying prediction, we performed a data-driven analysis across the entire sensor array for time windows shared across all tone duration conditions (0–150 ms). For each sensor, ${k}^{{\prime} }$-values from each tone duration condition were used to compute vector norm and vector angle to each hypothesis-derived orientation line. Comparison of these values against the null distribution derived from shuffled data (${k}_{{{\mathrm{shuff}}}}^{{\prime} }$) allowed us to identify sensor clusters showing significant effects ($p$ < 0.05, cluster-based permutation test, one-tailed; all *n* = 20; see Methods). Vector norm was significantly larger than expected under the null distribution (in the top 5th percentile of shuffled data) in widespread sensor clusters covering the whole head across all time windows (0–50 ms, 267 sensors, mean norm: 10.46 ± 0.67, $p$ < 0.001, ${d}_{{{\mathrm{cluster}}}}$ = 101; 50–100 ms: 260 sensors, mean norm: 10.45 ± 0.64, $p$ < 0.001, ${d}_{{{\mathrm{cluster}}}}$ = 76.9; 100–150 ms: 268 sensors, mean norm: 10.44 ± 0.67, $p$ < 0.001, ${d}_{{{\mathrm{cluster}}}}$ = 82.7; Supplementary Fig. 6), which is in line with the aforementioned SHI results (Supplementary Fig. 4). Analysis of vector angle towards the information line revealed a right central-lateral sensor cluster in the 0–50 ms window that was significantly smaller than expected under the null distribution (in the bottom 5th percentile of shuffled data; Fig. 5b; 4 sensors, mean angle: 0.017 ± 0.008, $p$ = 0.042, ${d}_{{{\mathrm{cluster}}}}$ = 2). Notably, these significant sensors completely overlapped with the right predictive processing cluster during the same time window (see Fig. 3a), reinforcing our conclusion that SHI underlying predictive computation operates over a fixed amount of information. For vector angle towards the duration line, an anterior-central sensor cluster where angles were significantly smaller than expected under the null distribution was present from 0–100 ms (Fig. 5c; 0–50 ms: 19 sensors, mean angle: 0.39 ± 0.029, $p$ = 0.008, ${d}_{{{\mathrm{cluster}}}}$ = 4.4; 50–100 ms: 15 sensors, mean angle: 0.4 ± 0.027 (SD), $p$ = 0.011, ${d}_{{{\mathrm{cluster}}}}$ = 3.8). This cluster had minimal overlap with predictive processing clusters (2/19 sensors for 0–50 ms; 1/15 sensors for 50–100 ms). Thus, positive evidence for the temporal bottleneck hypothesis exists, but appears spatially distinct from predictive neural activity.

### Neural and behavioral indices of sensory history integration are correlated across subjects

Finally, we investigated whether neural and behavioral effects of SHI are correlated across subjects. Similar to the above analysis, we first investigated sensors in predictive processing clusters and then conducted a data-driven analysis across the entire sensor array. To obtain a summary metric of neural SHI, we averaged ${k}^{{\prime} }$-values across the three tone duration conditions for each sensor and time window shared across all tone duration conditions (0–150 ms). To measure the influence of past tone pitches on the final tone pitch likelihood ratings (i.e., an influence of sensory history on a subject’s prediction responses), we used the $F$-statistic from the ${p}_{34}^{\ast }$ × ${p}_{34}$ interaction effect derived from a three-way repeated-measures ANOVA (factors: tone duration, ${p}_{34}^{\ast }$, ${p}_{34}$; dependent variable: final tone pitch likelihood ratings). This $F$-statistic quantifies how strongly the subject’s final tone pitch likelihood rating depends not only on the presented final tone pitch (${p}_{34}$), but also on the theoretically predicted final tone pitch given the previous sequence (${p}_{34}^{\ast }$).

**Fig. 6:**
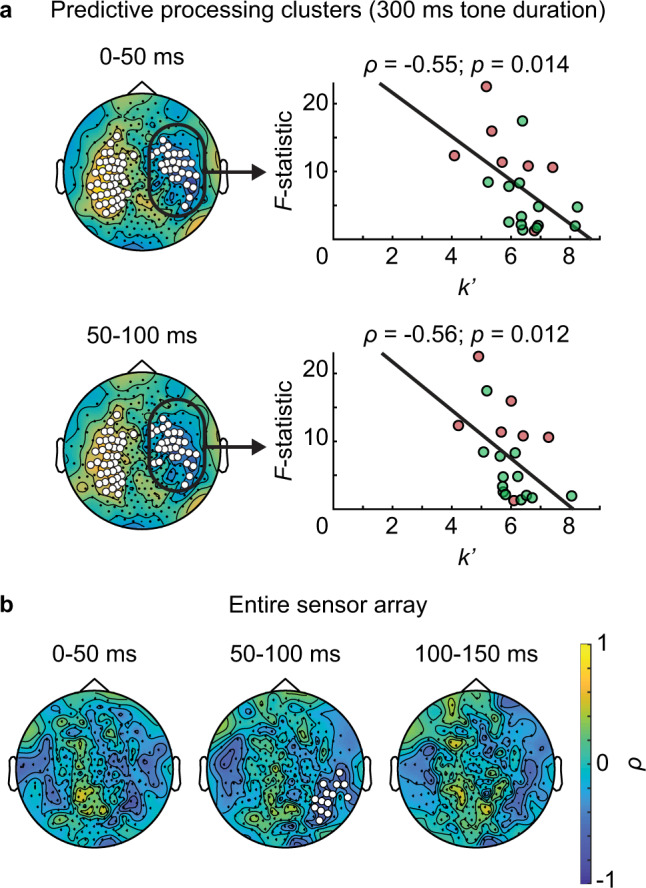
Number of neurally integrated tones correlates with behavioral indices of sensory history dependence across subjects (*n* = 20 participants). **a** Across-subject Spearman correlation of the number of preceding tones best explaining neuromagnetic activity ($k\mbox{'}$-values; averaged across-tone duration conditions and sensors in predictive processing clusters defined from the 300 ms condition) and $F$-statistics of the interaction effect between theoretically predicted final tone pitch (${p}_{34}^{\ast }$) and presented final tone pitch (${p}_{34}$; derived from a three-way repeated-measures ANOVA, factors: tone duration, ${p}_{34}^{\ast }$, ${p}_{34}$). In the scatter plots, each dot represents one subject. Red dots represent the seven subjects who received behavioral training, for whom data from the behavioral training session (with identical task paradigm as during the MEG session) were used. Results obtained using behavioral data from the MEG session for all subjects were qualitatively similar. Significant negative correlations (0–50 ms: $p$ = 0.014; 50–100 ms: $p$ = 0.012; all $p$ < 0.05 after FDR correction) were found for right sensor clusters at 0–50 ms and 50–100 ms. **b** Sensor-wise across-subject Spearman correlation of $k\mbox{'}$-values (averaged across-tone duration conditions for each sensor and TW) and $F$-statistics of the ${p}_{34}$ × ${p}_{34}^{\ast }$ interaction effect (derived from a three-way repeated-measures ANOVA, factors: tone duration, ${p}_{34}^{\ast }$, ${p}_{34}$) computed across the entire sensory array. White dots indicate significant sensor clusters ($p$ = 0.002, cluster-based permutation test, two-tailed).

In the first analysis, we assessed predictive processing clusters defined from the 300 ms condition (Fig. 3a). ${k}^{{\prime} }$-values were averaged across sensors in the predictive processing cluster for each subject, and a significant negative correlation with the behavioral SHI metric was found for the right predictive processing cluster in the first two time windows (0–50 ms: Spearman $\rho$ = −0.55, $p$ = 0.014; 50–100 ms: $\rho$ = −0.56, $p$ = 0.012; both $p$ < 0.05 after FDR correction; all *n* = 20; Fig. 6a). This suggests that subjects whose predictive neural activity integrates a smaller number of tones (to a minimum of four) exhibited a stronger behavioral prediction effect (i.e., a larger ${p}_{34}^{\ast }$ × ${p}_{34}$ interaction effect on final tone pitch likelihood ratings). Mechanistically, this finding can be explained by the consideration that integrating only the very last few tones produces exaggerated predictions for the upcoming final tone pitch (Fig. 1b): when ${p}_{34}^{\ast }$ is low, the subject’s prediction might be even lower, which would result in an excessive ${p}_{34}^{\ast }$ × ${p}_{34}$ interaction effect, producing an overshoot in the present metric for capturing predictive behavior.

Next, we performed a data-driven analysis across the entire sensor array. ${k}^{{\prime} }$-values in a right hemisphere sensor cluster were found to negatively correlate with behavioral SHI effects as measured by the ${p}_{34}^{\ast }$ × ${p}_{34}$ interaction effect (Fig. 6b; 50–100 ms: 14 sensors, average $\rho$: −0.61, $p$ = 0.002, ${d}_{{{\mathrm{cluster}}}}$ = 6.5; two-tailed cluster-based permutation test; *n* = 20). This sensor cluster partially overlaps with the predictive processing clusters, reinforcing our conclusion that SHI underlying predictive neural activity correlates with an individual subject’s predictive capability.

## Discussion

Effective prediction of future sensory input requires the nervous system to successfully integrate past sensory information. Variations in the rate with which the nervous system receives sensory information represent a fundamental computational challenge for prediction. Although such variations in sensory input rate are pervasive in natural settings^12,13^, it remains unclear how the nervous system achieves effective integration of sensory information and generates robust predictions in spite of such variation. Here, we investigated if neural integration windows subserving prediction are determined by a fixed temporal duration or a fixed amount of information (Fig. 1a). To adjudicate between these hypotheses, we systematically varied the presentation speed of stimulus sequences which capture the temporal statistical regularities of natural stimuli and assessed how the neural integration window depends on presentation speed. After confirming that subjects were able to make effective predictions across all presentation speeds, we found that the number of stimuli integrated in neural activity underlying prediction remains stable across a four-fold change in presentation speed (Fig. 5a). This indicates that neural integration windows subserving prediction are limited by an informational bottleneck and scale flexibly in time. Moreover, the length of neural integration windows in sensors carrying predictive information correlated with the behavioral sensory history integration effect across subjects.

Flexibly scaling neural integration windows discovered herein represent a core mechanism underlying predictive computation based on natural temporal regularities. Previous studies have shown successful sensory prediction despite varying sensory input rate at the behavioral level^21,22^, as well as adaptive neural responses to time-varying sensory input at the single-cell^23^ and population^14,21^ level. The connection of these findings to predictive computation, however, remained unclear. Using a novel and robust paradigm to tease apart predictive processing from instantaneous sensory processing, we were able to specifically probe the form of SHI underlying predictive computation and fill in the gap between these previously separate fields of inquiry.

Although predictive neural activity integrated over a fixed number of stimuli, analysis of the entire sensor array revealed that neural integration in frontal sensors acted according to the temporal bottleneck hypothesis (Fig. 5c). This frontal effect was spatially distinct from sensors in which neural activity contained predictive information. Our results therefore demonstrate that both temporally fixed and flexible integration windows govern SHI, but effects are located in spatially distinct sensor clusters, with flexible integration directly underlying predictive computation. The finding of parallel yet distinct modes of SHI beckons the question of how these integration processes are connected, which is an important topic for future investigation. One possibility is that temporally fixed integration windows in frontal areas act as temporary information storage^32^, which itself is not directly linked to prediction, but allows processes underlying prediction to selectively read out (or compare) relevant information originating in the past to optimize SHI subserving prediction.

Our study complements previous human neuroimaging work showing a hierarchy of temporal receptive windows involved in process memory^33^, as well as animal work showing a hierarchy of intrinsic timescales across the cortex^34^. At the lower end of this hierarchy, sensory cortices, with short temporal receptive windows (up to hundreds of milliseconds in humans), show rapid and transient responses to external stimulation^35^. Consistent with this finding, we observed instantaneous but short-lived responses to sensory input in the early sensory filter (Fig. 3b). At the other end of the hierarchy, higher-order association cortices are known to have long temporal receptive windows (spanning multiple seconds up to minutes in humans), enabling task-related accumulation of sensory information underlying decision making and working memory^35,36^. Here, we found that predictive information localized to anterior-lateral sensors overlying associative areas (Fig. 3a), and neural activity in these sensors contains a continuous buildup of predictive activity spanning multiple tones over extended periods of time (Fig. 3b). Importantly, the present study empirically connects this rich prior literature on a hierarchy of timescales with predictive computation (but see ref. ^37^ for a computational approach), revealing that predictive neural activity directly emerges from flexible neural integration of sensory history with an extended time scale.

The respective contributions of the left and right hemisphere to sensory history integration and predictive computation require further investigation. We found bilateral predictive neural activity (Fig. 3a and Supplementary Fig. 1) and bilateral SHI. Significant evidence for the information bottleneck hypothesis was also found bilaterally (Fig. 5a, b). Yet, the length of neural integration windows correlated strongly with behavior only for the right predictive processing clusters (Fig. 6). Taken together, these results suggest that both the left and right hemispheres participate in SHI and predictive processing.

It is important to consider whether the present neural prediction effects and their dependence on sensory history integration relate to the well-known neural adaptation effects. Despite being an extensively studied topic in neuroscience^38^, adaptation remains a relatively broad term capturing a variety of context- or history-dependent neural response dynamics. The distinction between neural adaptation and prediction effects is subtle, with terminology differing for micro (i.e., single cell) vs. macro (i.e., population level) recordings, mechanistical vs. functional models, and computational vs. cognitive literature^39^. Unsurprisingly, both concepts are tightly linked and increasing evidence^23,40^ points towards adaptation as a single-neuron correlate of mismatch negativity, a cortical potential difference between a deviant and a standard stimulus in repetition-based paradigms classically used in prediction studies. The predictive processing framework provides a unifying conception since it interprets repetition and expectation suppression, neural implementations of adaptation and prediction, as manifestation of prediction error signals on different temporal and spatial scales^41,42^. However, adaptation alone is unable to account for multiple prediction-related mismatch negativity findings, such as increased mismatch negativity in response to stimulus omissions and unpredicted versus predicted deviant tones^43^. Specifically, (stimulus specific) adaptation depends on the frequency with which a stimulus is presented, whereas mismatch negativity relies mainly on transitional probabilities and not stimulus frequency itself^43^. Furthermore, neural adaptation is known to influence evoked neural responses comparatively early (from 50 ms onward), whereas effects of prediction are visible in later responses (after 100 ms)^44,45^. Given the extended duration of the current prediction and history tracking effects, it is therefore unlikely that these can be explained solely by adaptation. In addition, adaptation effects are mainly found in primary^23^ and nonprimary^40^ sensory cortex areas, and nonprimary sensory areas show surprise-based enhancement of neural responses that cannot be explained by adaptation^46^. The wide spatial extent of the present prediction and sensory history integration findings, along with the lack of prediction effects in neural recordings focusing on early sensory processing, underscores that adaptation alone cannot explain the current findings. This is further supported by the stability of the prediction-related behavioral and neural results across the first and second halves of the experiment (Supplementary Fig. 2). Finally, in contrast to oddball tasks, the present stimuli set does not rely on repeated presentation of identical stimuli (the classic approach to induce stimulus-specific adaptation), rendering our paradigm less reliant on adaptation effects. This is especially true for sequences with low beta level, which nonetheless produce prominent prediction and sensory history tracking effects (Supplementary Fig. 7). A clean distinction between adaptation and prediction or SHI effects, however, requires specific experimental manipulations (e.g., systematic control for item frequency) and use of conditions known to distinguish between adaptation and prediction (e.g., omission responses^47^).

Interpretation of the present results requires consideration of some limitations. First, the flexibly scaling integration windows observed herein likely break down at extreme stimulus presentation rates. We employed a limited range of tone durations, selected to be conducive for human task performance. Future studies will determine at which sensory input rates flexible integration breaks down. Second, the present study does not specify the anatomical sources underlying prediction and SHI effects. This is mainly because a reliable and temporally stable source localization of the corresponding neural generators is impeded by the use of non-baseline-corrected activity, as accumulating neural activity increasingly affects source localization precision over the course of sequence presentation. Future work employing intracranial recordings will shed light on the anatomical sources giving rise to the integrative and predictive neural activity uncovered in the present work. Third, additional work remains to be carried out to fully reveal the computational mechanisms underlying subjects’ predictive behavior. While the present findings and our earlier study^25^ show that the sensory history integration carried in neural activity follows a weighted-linear-sum form (with weights differing by temporal position, see Fig. 7 in ref. ^25^) and directly contributes to predictive neural activity, whether such a computation fully captures subjects’ predictive strategy as evidenced in their behavior remains to be tested. This question can be addressed by conducting model-fitting and model-comparison on subjects’ single-trial behavior; such an endeavor would also illuminate whether subjects use simple heuristics (e.g., continue the recent past) or a more complex strategy (e.g., involving nonlinear extrapolation, but note that the theoretically optimal prediction, ${p}_{34}^{\ast }$, embodies a linear computation) in forming predictions.

Prediction about future sensory input has been commonly studied through statistical learning paradigms involving repeated presentations of an item (such as variations of the oddball paradigm^48^) or a sequence with certain transition probabilities embedded (e.g., frequent ABC^49^). These paradigms offer precise control of various statistical regularities (e.g., item frequency, alternation frequency, transition probability), which in turn allows for a systematic analysis of the neural processes related to sequence dependence and its dissection into local and global components^29,45,50^. The present paradigm departs from these earlier paradigms by generating predictive content with a continuous value^25^ that relies on more complex statistical regularities going beyond repetition. This approach allows us to specifically probe predictive computation based on temporal statistical regularities that are inherent to and prevalent in natural stimuli—a brain process that remains poorly understood. Because the brain evolved in the natural environment where effective predictions about future stimuli is crucial to survival^10^, understanding predictive processing of natural statistical regularities sheds light on a fundamental brain function. Future studies will further illuminate how neural mechanisms underlying predictions based on these naturalistic statistical regularities compare with those serving predictions in classic statistical learning paradigms.

In conclusion, the present work demonstrates that neural activity integrates past sensory information both over fixed amounts of information and fixed durations, and that these processes are spatially separated. However, integration underlying prediction of upcoming sensory input mainly operates over a fixed amount of information and is limited by an informational bottleneck. Flexible sensory history integration enables precise prediction in the face of varying stimulus input rates and represents a fundamental mechanism underlying humans’ ability to make robust predictions in natural environments.

## Methods

### Participants

Twenty six healthy right-handed subjects with normal hearing took part in the experiment. Seven subjects were prescreened for behavioral performance in a training session prior to the MEG recording. Six subjects were excluded due to either poor performance (i.e., not using the full range of the rating scale) or excessive MEG artifacts, yielding a final group of 20 subjects (11 females; mean age 25.0, 19–34 y). A reduced version of the behavioral and neural prediction results obtained from the 300 ms tone duration condition of this dataset was previously reported as an independent replication of main results in ref. ^25^. The study was approved by the Institutional Review Board of the National Institute of Neurological Disorders and Stroke (protocol #14-N-0002). All subjects provided written, informed consent.

### Experimental stimuli

The present study employed naturalistic auditory tone sequences with pitch fluctuations exhibiting statistical regularities similar to those prevalent in natural stimuli^26,27^. Specifically, each sequence consisted of 34 concatenated pure tones presented without temporal overlap or gap. Within the same sequence, tone pitches were temporally dependent upon each other (i.e., autocorrelated over time), allowing for the prediction of future tone pitches as a function of previously presented tone pitches. The degree of autocorrelation within each sequence was determined by *β*, which defines the relationship between the frequency of pitch fluctuations over time and the power of fluctuations at the respective frequency, such that *P* ≈1 / f ^β^ (i.e., the temporal power spectrum of pitch fluctuation). Consequently, a *β* of 0 means that pitch values between any two time points are uncorrelated, while a high *β* implies temporally adjacent tone pitches are positively dependent on one another. Further details regarding the tone sequence creation are described in detail in ref. ^25^. The present auditory tone sequences were constructed with three levels of autocorrelation strength *β*: 0.5, 0.99, and 1.5.

In accordance with ref. ^25^, each tone series was scaled such that its pitches ranged from log(220) to log(880). Tone series were discretized so that each tone was assigned to one of 25 values evenly spaced on the log scale with semitone distance. A circulant embedding algorithm^51^ was used to create nine unique 33-element long series, three for each *β* level:

1 $${x}_{\beta ,i}=\left[{x}_{1},\ldots ,{x}_{33}\right],\beta \in \left\{0.5,0.99,1.5\right\},1\le i\le 3$$

where each element ${x}_{j}$ of ${x}_{\beta ,i}$ is taken to represent the pitch of the j^th^ tone in the sequence. Importantly, the choice of autocorrelation strength *β* lies within the range of natural acoustic signals, for which *β* commonly ranges between 0 and 2^26^. The full set of tone sequences can be downloaded at:

https://med.nyu.edu/helab/sites/default/files/helab/Baumgarten_etal_stim_wav_files_and_figs.zip

All tone sequences converged on an identical penultimate (33rd) tone pitch (440 Hz), ${p}_{33}$. This allowed us to disentangle sensory processing of ${p}_{33}$ from predictive processing relying on ${p}_{1-32}$. Specifically, since ${p}_{33}$ was held constant across trials, it can be excluded from a regression which seeks to explain differences in neural activity during the presentation of the 33rd tone as a function of the previous tone sequence and the predicted upcoming tone pitch based on it.

For each tone sequence, a specific theoretically predicted final (34th) tone pitch (${p}_{34}^{\ast }$; see refs. ^10,25^ for further details) was computed, representing the optimally fitting final tone pitch given the pitch information provided by the first 33 tones. Nine unique sequences (Fig. 1b) were selected to represent all combinations of temporal autocorrelation level *β* (0.5, 0.99, 1.5) and three bins of theoretically predicted final tone pitch (${p}_{34}^{\ast }$: low [370 Hz, 392 Hz], medium [440 Hz], high [494 Hz, 523 Hz]).

To probe subjects’ ability to predict the final tone pitch, the actually presented 34th tone of each sequence (${p}_{34}$) was independently drawn from one of six possible pitches located four [349 Hz/554 Hz], eight [277 Hz/699 Hz], or twelve [220 Hz/880 Hz] semitone steps below/above the mean pitch value of 440 Hz. Consequently, for a listener who can optimally extract the sequence information provided by the temporal autocorrelation within a given tone sequence, the tone pitch distance between ${p}_{34}$ (i.e., the presented final tone) and ${p}_{34}^{\ast }$ (i.e., the theoretically predicted final tone) should determine if ${p}_{34}$ is considered likely or unlikely given the information provided by ${p}_{1-33}$.

Identical tone sequences were presented in different tone duration conditions, comprising short (150 ms per tone/5.1 s per sequence), medium (300 ms/10.2 s), or long (600 ms/20.4 s) tone duration. The medium condition was used as the representative condition to determine sensor clusters of interest in later analyses.

In total, nine unique sequences (3 *β* levels × 3 ${p}_{34}^{\ast }$) × 3 tone durations constituted 27 distinct auditory sequences. Each distinct sequence was presented once within each of 12 blocks in random order, resulting in a total of 324 trials per subject.

### Paradigm

Auditory tone sequences were presented with a sampling frequency of 44,100 Hz using the PsychPortAudio function of the Psychophysics Toolbox^52^ in MATLAB (The Mathworks Inc., Natick/MA, USA) and specialized MEG-compatible ear tube (Etymotic ER-3 Insert Headphones). The plastic tubing from the transducer to the earpiece had a speed-of-sound delay of approx. 10 ms, which was corrected for in MEG data analyses.

Each trial began with the presentation of a blank screen (duration: 0.5 s), followed by the central presentation of a fixation point (0.7 s, Fig. 1c). Next, tone sequences were presented (5.1/10.2/20.4 s) while the fixation point remained on the screen. Subjects were instructed to fixate on the fixation point during its entire presentation to minimize eye movements. Next, a blank screen was presented for 0.4 s after which subjects rated how likely the final tone pitch was given the previously presented tone sequence. In other words, subjects rated how well ${p}_{34}$ agreed with the overall pattern of tone pitches present in the preceding tone sequence. Final tone pitch likelihood ratings were given on a scale of 1 (least expected) to 5 (most expected) within a response window of 5 s and without feedback. Next, subjects rated the trend strength (i.e., autocorrelation) of the presented tone sequence within a response window of 5 s. The trend strength ratings were given on a scale from 1 (most random) to 3 (most trend-like). Feedback about the performance in the trend strength rating task was presented visually for 2 s after entry of both behavioral responses. The feedback indicated which trend strength rating had been entered by the subject, what the true trend strength of the sequence was, and whether the subject’s trend strength rating was correct, close to correct (off by one level), or incorrect (off by two or more levels). Responses were entered using two separate button boxes, with final tone pitch likelihood ratings being entered by the left hand and trend strength ratings being entered by the right hand.

Across the entire experiment, trials were split into 12 blocks with 27 trials each. Subjects were given the option to take self-terminated rest periods after each block. Head position within the MEG sensor array was measured after each block by means of coils placed on the left and right preauricular points and the nasion. Subjects self-corrected their head position in order to closely match the position at the start of the experiment^25^. The entire experiment lasted for approximately 3 h including setup time.

### Analyses of prediction task performance

Subjects’ final tone pitch likelihood ratings indirectly allow us to investigate if subjects are able to extract the information provided by the preceding tone sequence and correctly predict the future final tone’s pitch. If this is the case, final tone pitch likelihood ratings should be a function of both ${p}_{34}$ and ${p}_{34}^{\ast }$, since ${p}_{34}^{\ast }$ represents which final tone is most likely to be presented given the preceding tone sequence. Final tone pitch likelihood ratings were analyzed by means of within-subject effects of a three-way repeated-measures ANOVA at the group level with factors: tone duration, ${p}_{34}^{\ast }$, and ${p}_{34}$. To analyze if final tone pitch likelihood ratings were higher for trials with low ${p}_{34}$ when the preceding tone sequence converged on a low ${p}_{34}^{\ast }$ instead of a high ${p}_{34}^{\ast }$ (and vice versa for trials with high ${p}_{34}$), paired $t$-tests across subjects were used. To determine prediction effects per tone duration condition, final tone pitch likelihood ratings were analyzed by means of a two-way repeated-measures ANOVA at the group level with factors: ${p}_{34}^{\ast }$ and ${p}_{34}$. In addition, a three-way (factors: ${p}_{34}$, ${p}_{34}^{\ast }$, tone duration) repeated-measures ANOVA was performed at the single-subject level to resolve an $F$-statistic for the interaction effect of ${p}_{34}^{\ast }$ and ${p}_{34}$ on final tone pitch likelihood ratings, which was used as an individual index quantifying the history dependence of final tone pitch likelihood ratings across all tone duration conditions. Sphericity was tested with Mauchly’s test and sphericity violations were corrected by means of Greenhouse-Geisser correction.

For the seven subjects who underwent behavioral prescreening, results reported in Figs. 2 and 6 used their behavioral data from the training session, ensuring that assayed task performance was always based on the initial task encounter. For the remaining subjects who performed the task only during MEG recording, behavioral data from the MEG session were used. Data calculation based on behavioral data from the MEG recording session for all subjects produced qualitatively similar results for all analyses.

### MEG data acquisition and preprocessing

Whole-head neuromagnetic activity was recorded during the task with a 275-channel CTF MEG scanner (VSM MedTech, Coquitlam, BC, Canada). Three malfunctioning sensors were excluded from analysis (leaving 272 channels in total). Scans were completed at a sampling rate of 600 Hz, with an anti-aliasing filter applied at <150 Hz. Custom-written MATLAB scripts and the Fieldtrip Toolbox (http://www.fieldtriptoolbox.org/;^53^) were used for all preprocessing and analyses steps. MEG data from each block were demeaned and detrended. No high-pass filter was applied, in order to retain low-frequency information (see refs. ^25,29^). A Butterworth band-stop filter for 58–62 Hz and 118–122 Hz was applied to remove line noise. Independent component analysis was applied on filtered data to remove artifacts due to eye blinks and ocular motion, heartbeat, breathing, and movement-related slow drift.

### Computing neuromagnetic correlates of prediction

We tested whether neural activity during the presentation of ${p}_{33}$ contains information about the theoretically predicted upcoming final tone pitch (${p}_{34}^{\ast }$). To this end, a linear regression was performed separately for each tone duration condition. First, neuromagnetic activity during presentation of ${p}_{33}$ was segmented into nonoverlapping time windows of 50 ms duration (e.g., 0–50 ms, 50–100 ms, …). This resulted in a different number of time windows entering the regression analysis for the three tone duration conditions (i.e., 3, 6, and 12 time windows for the 150, 300, 600 ms tone duration conditions). Sensor-wise non-baseline-corrected neuromagnetic activity was averaged within a time window, resulting in an estimate for neuromagnetic activity per sensor, time window, and trial. For each subject, neuromagnetic activity *N* at each sensor *s*, time window *w*, and trial *n* during ${p}_{33}$ was linearly regressed onto ${p}_{34}^{\ast }$:

2 $${N}_{s,w,33,n}={\beta }_{0,s,w}^{\ast }+{\beta }_{1,s,w}^{\ast }{p}_{34,n}^{\ast }+{\varepsilon }_{s,w,33,n}$$

Any neuromagnetic activity associated with the processing of ${p}_{33}$ could be assumed to remain constant across trials, since the pitch of the 33rd tone was always 440 Hz, such that this term could be excluded from the regression analysis. ${\beta }_{1,s,w}^{\ast }$ describes how neuromagnetic activity during ${p}_{33}$ depends on ${p}_{34}^{\ast }$. At the group level, all subjects’ ${\beta }_{1,s,w}^{\ast }$ regression weights were submitted to a one-sample $t$*-*test against 0, yielding an uncorrected $t$-value for each sensor and time window. Sensor-wise $t$-values then underwent permutation-based cluster correction. Spatially contiguous sensors exhibiting a significant effect were defined by comparison to a permutation-derived null distribution. For each subject, data were permuted by randomly shuffling the across-trial dependence between the dependent variable (MEG activity during ${p}_{33}$) and the independent variable (${p}_{34}^{\ast }$). Clusters were defined as significant if their summary statistic ($\left|\sum t\right|$, where $t$-values had the same sign) was in the top 2.5th percentile of shuffled data, corresponding to a two-tailed test at $p$ < 0.05. Significant sensor clusters are referred to as predictive processing clusters.

To visualize neuromagnetic activity during ${p}_{33}$ as a function of ${p}_{34}^{\ast }$, trials were binned into low, medium, and high ${p}_{34}^{\ast }$ for each subject. For each timepoint, neuromagnetic activity was averaged across each group of trials for sensors within a predictive processing cluster, and then averaged across subjects.

The time course of predictive information buildup in slow arrhythmic neural activity over the course of the tone sequence presentation was investigated by comparing time-resolved neural activity time-locked to tone presentation between sequences converging on low vs. high ${p}_{34}^{\ast }$. Sensor-wise time-locked neural data were low-pass filtered at 35 Hz and subtracted by the mean amplitude from a 500 ms time window preceding the first tone. For each subject and tone duration condition, neural data was averaged across all trials converging on low or high ${p}_{34}^{\ast }$, respectively. Group-level comparison of time-locked data was performed for all samples ranging from the offset of the first tone to the start of the response window by means of a one-sample $t$*-*test yielding an uncorrected $t$-value for each sample. To account for multiple comparisons, $t$-values were statistically assessed with a cluster-based nonparametric randomization approach^54^. To differentiate the effects of early sensory processing from predictive processing, time-locked neural activity was calculated using two different methods. First, predictive processing was investigated using predictive processing clusters carrying significant predictive information about ${p}_{34}^{\ast }$ for each tone duration (defined using 50–100 ms window for 300 ms, and 100–150 ms window for 150 and 600 ms, so all clusters are in the right hemisphere; see Fig. 3 and Supplementary Fig. 3). Early sensory processing was investigated using a spatial filter focusing on early sensor processing, defined as follows.

### Definition of spatial filters focusing on early sensory processing

The M100 response represents a common auditory functional localizer^30^ known to underlie the initial stages of auditory stimulus processing^31^, which originates in Heschl’s gyrus and primary auditory cortex. Separately for each subject and tone duration condition, sensor-wise responses time-locked to each tone were first computed for the M100 time window (75–125 ms after tone presentation) for each of the 34 tones within a sequence, yielding a time-locked response to auditory stimulation. Next, this tonal response was averaged across tones and across trials. Resulting ERF time course values were squared and averaged across the M100 time window. Based on these average M100 amplitude values, the relative contribution of each sensor towards the overall signal was determined. The resulting sensor weights were multiplied with the nonsquared neuromagnetic data and subsequently averaged across sensors, yielding a weighted spatial filter for each subject. Neural activity projected through these spatial filters highlight tone processing in auditory cortex areas, referred to as early sensory filter.

### Computing neuromagnetic correlates of SHI

Effective prediction requires accumulation and integration of past sensory history information (SHI). To assess SHI, we analyzed the dependence of neural activity during the second half of a tone sequence on the pitch of preceding tones (Fig. 4). ${p}_{33}$ was excluded since it was constantly presented at 440 Hz and ${p}_{34}$ was excluded since its pitch was determined independently of the preceding tone sequence. Analyses were performed separately for each tone duration condition, yielding 108 trials per analysis. Similar to the prediction analysis, sensor-wise non-baseline-corrected neuromagnetic activity was averaged across time using nonoverlapping windows of 50 ms duration. Next, the neuromagnetic activity $N$ at each sensor $s$, time window $w$, and trial $n$ during the presentation of the current tone $i$ (16 ≤ $i$ ≤ 32) was linearly regressed onto the pitch $p$ of the current tone and ${k}^{{\prime} }$ previous tones as follows:

3 $${N}_{s,w,i,n}={\beta }_{0,s,w}+\mathop{\sum }\limits_{k=0}^{k=k{\prime} }{\beta }_{k+1,s,w}{p}_{i-k,n}+{\varepsilon }_{s,w,i,n}$$

The parameter ${k}^{{\prime} }$ represents how many previous tones explain MEG activity at the current timepoint. We tested 16 models with 1–16 regression terms, corresponding to ${k}^{{\prime} }$-values ranging from 0 (current tone pitch) to 15 (current tone pitch and 15 previous tones).

To determine which model (i.e., which value of ${k}^{{\prime} }$) best accounts for neuromagnetic activity, a six-fold cross-validation approach was used. Each subject’s data were split into six folds, with each fold being used as a test fold once and as a training fold in the remaining five runs. The allocation of trials to a fold was balanced for each of the nine unique tone sequence (i.e., determined by ${p}_{1-33}$). Since 12 repetitions for each unique tone sequence were presented for each tone duration, two randomly selected repetitions for each unique tone sequence were allocated to each fold.

First, regression coefficients for each sensor, time window, and linear model were calculated based on the training set. The resulting regression coefficients were then used to calculate the current model’s prediction of MEG activity in the test set. Model selection was based on the minimized sum of squared errors. The winning ${k}^{{\prime} }$-value indicates how many previous tones in a sequence best explain the recorded MEG activity per sensor, time window, and tone duration. After computing ${k}^{{\prime} }$ for all folds, we averaged the ${k}^{{\prime} }$-values across folds to calculate the final ${k}^{{\prime} }$-value computed for experimental data for each subject, at each sensor and time window.

Statistical significance of sensor clusters showing SHI effects was assessed by means of a nonparametric permutation test comparing $k^{\prime}$ against a null distribution based on shuffled tone order. By shuffling tone order within each sequence, the temporal dependence between neural activity and tone sequence was destroyed, in line with the null hypothesis that there is no systematic integration of previously presented tones. To assess significance of the (across-subject) average ${k}^{{\prime} }$-values against this null hypothesis, we repeated the abovementioned cross-validation regression analyses but with randomly permutated tone order within each sequence in the training set, while leaving tone order in the test set unperturbed. The order of the first 32 tones in the training set was shuffled, with the exception that the value of the current ($i$-th) tone pitch was kept the same as in the original sequence. Thus, information about current tone pitch was retained, whereas information about tone sequence history was destroyed. This procedure was repeated 100 times for each dataset (i.e., for each of the six folds, separately for each tone duration condition and each subject). Tone pitch order was shuffled differently for each of the nine unique tone sequences, but was kept constant across the 12 repetitions of each unique tone sequence. Importantly, to enable a valid comparison across-tone duration conditions, shuffle order for each unique sequence was preserved across tone duration conditions. Tone order was shuffled anew for every test/training fold combination. Identical to the computation of $k^{\prime}$-values, the same six-fold cross-validation technique was applied to extract the optimal $k^{\prime}$-value computed for shuffled data (${k}_{{{\mathrm{shuff}}}}^{{\prime} }$) for each sensor and time window in each tone duration-specific dataset per subject. This yielded a shuffled null distribution of 600 $k^{\prime}$-values per sensor, time window, tone duration condition, and subject. ${k}_{{{\mathrm{shuff}}}}^{{\prime} }$-values were subsequently averaged across folds to yield 100 shuffled values per subject.

Effects at the group level were assessed by first averaging $k^{\prime}$-values across subjects for each sensor and time window within each tone duration separately. Group-level effects of SHI in neuromagnetic activity were compared against the null hypothesis that there is no systematic SHI across a tone sequence. To create the null distribution against which to compare group-level effects of SHI in neuromagnetic activity, repeated samples (with replacement) were drawn 1000 times from the null distribution of each subject, with each draw subsequently averaged across subjects. This formed a null distribution of 1000 across-subject-averaged ${k}_{{{\mathrm{shuff}}}}^{{\prime} }$-values. This shuffling procedure instantiates sampling under the null hypothesis since ${k}_{{{\mathrm{shuff}}}}^{{\prime} }$-values reflect the magnitude of $k^{\prime}$ that can be expected due to unspecific noise, since the shuffled tone order was not presented to the subject and therefore neuromagnetic activity cannot contain genuine SHI of the shuffled tone sequence. An uncorrected $p$-value was assigned to each sensor for each time window, calculated as the proportion of ${k}_{{{\mathrm{shuff}}}}^{{\prime} }$-values larger than or equal to the $k^{\prime}$-value. Subsequently, uncorrected $p$-values were used to define clusters for the cluster correction analysis. Clusters were considered significant if their cluster statistics calculated for experimental data lied in the top 5th percentile of shuffled data, corresponding to a one-tailed test at $p$ < 0.05.

### Comparing sensory history integration across tone duration conditions

Comparing estimates of SHI across tone duration conditions allows us to test if the brain integrates sensory history over a fixed temporal duration or over a fixed amount of information (Fig. 1a). $k^{\prime}$-values for sensors in predictive processing clusters were used to estimate neurally integrated tone information relevant for prediction (Fig. 4). Analysis was performed separately for each sensor cluster (i.e., left vs. right hemisphere) in time windows from 0–150 ms after tone onset. For each sensor and time window, ${k}^{{\prime}}$-values were averaged across subjects separately for each tone duration condition. Group-averaged $k^{\prime}$-values were projected into a three-dimensional coordinate system, where each axis was defined as $k^{\prime}$-value for a specific tone duration condition. To differentiate between the abovementioned hypotheses, two hypothesis-derived orientation lines were projected on the coordinate system. Integration over a fixed duration corresponds to a line with the slope ${{k}^{{\prime} }}_{150{msTD}}=2{{k}^{{\prime} }}_{300{msTD}}=4{{k}^{{\prime} }}_{600{msTD}}$ (red line in Figs. 4 and 5); integration over a fixed amount of information corresponds to a line with the slope ${{k}^{{\prime} }}_{150{msTD}}={{k}^{{\prime} }}_{300{msTD}}={{k}^{{\prime} }}_{600{msTD}}$ (blue line in Figs. 4 and 5).

SHI per sensor $s$ and time window $w$ can now be conceptualized as a vector ${\vec{{\boldsymbol{u}}}}_{{\boldsymbol{s}},{\boldsymbol{w}}}$ in the 3-D $k^{\prime}$-space, spanning from the point of origin to the coordinates determined by the $k^{\prime}$-values in the ${x}_{s,w}$ (150 ms), ${y}_{s,w}$ (300 ms), and ${z}_{s,w}$ (600 ms) dimension. For each vector ${\vec{{\boldsymbol{u}}}}_{{\boldsymbol{s}},{\boldsymbol{w}}}$, we computed norm (i.e., vector magnitude measured from the point of origin) and angle to the respective orientation lines. Whereas norm indicates the overall number of integrated tones, angle indicates how close the vector is to the respective orientation line. Vector norm was computed as:

4 $${\rm{||}}{\vec{{\boldsymbol{u}}}}_{{\boldsymbol{s}},{\boldsymbol{w}}}{\rm{||}}=\sqrt{{x}_{s,w}^{2}{+y}_{s,w}^{2}+{z}_{s,w}^{2}}$$

Vector angle ${\theta }_{s,w}$ was defined as the angle between ${\vec{{\boldsymbol{u}}}}_{{\boldsymbol{s}},{\boldsymbol{w}}}$ and the vector determined by the respective orientation line, i.e., either duration (${\vec{\boldsymbol{v}}}_{{\boldsymbol{dur}}}=({\mathrm{4,2,1}})$) or information line (${\vec{\boldsymbol{v}}}_{{\boldsymbol{info}}}=({\mathrm{1,1,1}})$). Vector angle (Eq. (7)) was computed based on the cross-product (Eq. (5)) and the dot-product (Eq. (6)):

5 $$\vec{{\boldsymbol{u}}}\times \vec{{\boldsymbol{v}}}=({u}_{y}{v}_{z}-{u}_{z}{v}_{y},{u}_{z}{v}_{x}-{u}_{x}{v}_{z},{u}_{x}{v}_{y}-{u}_{y}{v}_{x})$$

6 $${\vec{{\boldsymbol{u}}}}\cdot \vec{{\boldsymbol{v}}}={u}_{x}\times {v}_{x}+{u}_{y}\times {v}_{y}+{u}_{z}\times {v}_{z}$$

7 $${\theta }_{s,w}=\tan ^{-1}({\rm{||}}\vec{{\boldsymbol{u}}}\times \vec{{\boldsymbol{v}}}{\rm{||}},{\vec{{\boldsymbol{u}}}}\cdot \vec{{\boldsymbol{v}}})$$

and expressed in radians. To investigate the effect of SHI underlying prediction, norm and angle were averaged across all sensors within a specific predictive processing cluster, (${||}{\vec{{\boldsymbol{u}}}}_{{\boldsymbol{pred}},{\boldsymbol{w}}}{||}$, ${\theta }_{{pred},w}$).

As can be seen in Fig. 5, $k^{\prime}$-values generally cluster closer to the information line compared to the duration line. However, ${k}_{{{\mathrm{shuff}}}}^{{\prime} }$-values likewise generally cluster closer to the information line, because shuffling tone order within a sequence should yield similar ${k}^{{\prime} }$-values across all tone durations. To account for this, we compared $k^{\prime}$-values against ${k}_{{{\mathrm{shuff}}}}^{{\prime} }$-values, which allows us to statistically determine if $k^{\prime}$-values are closer to the respective orientation line than chance. Significance of both vector norm and angle was assessed by means of a nonparametric permutation test comparing $k^{\prime}$-values against a null distribution constructed from ${k}_{{{\mathrm{shuff}}}}^{{\prime} }$-values. Computation of the null distribution was performed as specified above. Effects of vector norm were considered significant if their cluster statistic (summed norm) lies in the top 5th percentile of shuffled data, corresponding to a one-tailed test at $p$ < 0.05. Effects of vector angle were considered significant if their cluster statistic (summed angle) lies in the bottom 5th percentile of shuffled data, corresponding to a one-tailed test at $p$ < 0.05.

To allow for an easier visualization of data in relation to the two hypotheses, we additionally projected $k^{\prime}$-values onto a two-dimensional plane (${k^{\prime}}_{2-D}$), defined by the two hypothesis-derived orientation lines. To this end, we computed the cross-product (Eq. (5)) of $\vec{\boldsymbol{v}}_{\mathbf{dur}}$ and ${\vec{\boldsymbol{v}}}_{{{{\mathbf{info}}}}}$, resulting in the vector ${\vec{{\boldsymbol{N}}}}_{{{{\mathbf{dur}}}}{{-}}{{{\mathbf{info}}}}}$ normal to a plane spanned between ${\vec{\boldsymbol{v}}}_{{{{\mathbf{dur}}}}}$ and ${\vec{\boldsymbol{v}}}_{{{{\mathbf{info}}}}}$. Next, we projected both $k^{\prime}$- and ${k}_{{{\mathrm{shuff}}}}^{{\prime} }$-values for each sensor and time window from the 3-D space to the nearest point on this plane (Eq. (8)).

8 $${k^{\prime}}_{2-D} = {k^{\prime}}_{3-D} + | \left(\right.0 - \sum ({k^{\prime}}_{3-D} . * {\vec{\boldsymbol{N}}}_{{\mathbf{dur}}-{\mathbf{info}}})/ \sum ({\vec{\boldsymbol{N}}}_{{{\mathbf{dur}}-{\mathbf{info}}}} . * {\vec{\boldsymbol{N}}}_{{{\mathbf{dur}}-{\mathbf{info}}}}) | * {\vec{\boldsymbol{N}}}_{{{\mathbf{dur}}-{\mathbf{info}}}}$$

$k^{\prime}$- and ${k}_{{{\mathrm{shuff}}}}^{{\prime} }$-values projected to this 2-D space were plotted, and vector norm and angle in the 2-D space were computed in the same manner as in the 3-D space.

Effects of vector norm and angle were also analyzed across the entire sensor array. Vector norm and angle were computed for each sensor and for time windows from 0–150 ms. Sensor- and time window-specific norm and angle derived from $k^{\prime}$-values were compared against a null distribution computed from ${k}_{{\mathrm{shuff}}}^{\prime}$-values. Cluster correction was applied to determine significant sensor clusters.

### Correlating behavioral and neural sensory history tracking effects across subjects

Finally, we tested whether neural and behavioral effects of SHI correlated across subjects. To increase analytic power and to assess general task performance, we computed correlations for data reflecting general effects across tone durations, resulting in one data point per subject. For each subject, we used the $F$-statistic for the interaction effect of ${p}_{34}^{\ast }$ and ${\rm{p}}_{34}$ derived from a three-way repeated-measures ANOVA (factors: tone duration, ${p}_{34}^{\ast }$, ${p}_{34}$) to indicate how strongly sensory history affected a subject’s prediction responses. Likewise, we averaged $k^{\prime}$-values across tone duration conditions for each sensor and time window present in all tone duration conditions (i.e., 0–150 ms).

First, we investigated predictive processing clusters. Individual $k^{\prime}$-values were averaged across all sensors within each respective predictive processing cluster determined for the 300 ms condition. Spearman correlation was calculated between the resulting $k^{\prime}$-values and $F$-statistics across subjects. $p$-values were corrected for multiple comparisons by means of false discovery rate (FDR).

Next, we conducted a sensor-wise analysis across the entire sensor array to see if $k^{\prime}$-values correlated with behavioral effects of history dependence in sensors outside of predictive processing clusters. To this end, we computed across-subject Spearman correlations between $k^{\prime}$-values (for each sensor and time window) and $F$-statistics. To correct for multiple comparisons, a group-level null distribution based on ${k}_{{{\mathrm{shuff}}}}^{{\prime} }$-values was constructed. For this, each of the 100 repetitions of individual ${k}_{{{\mathrm{shuff}}}}^{{\prime} }$-values was averaged across tone duration conditions for each sensor and time window. Next, one repetition per subject was chosen randomly, which was repeated 1000 times to construct a shuffled distribution containing 1000 random draws of ${k}_{{{\mathrm{shuff}}}}^{{\prime} }$-values for each subject. The resulting ${k}_{{{\mathrm{shuff}}}}^{{\prime} }$-values were then correlated with $F$-statistics to create a null distribution of correlation values for each sensor and time window. Sensor clusters showing a significant correlation were compared against the null distribution. Clusters were defined as significant if their cluster statistics ($\left|\sum \rho \right|$, where $\rho$-values had the same sign) calculated for experimental data lie in the top 2.5th percentile of shuffled data, corresponding to a two-tailed test at $p$ < 0.05.

### Cluster correction

Cluster-based permutation testing^54^ was used to define clusters of spatially contiguous sensors showing a significant prediction, SHI, or correlation effect and to correct for multiple comparison across sensors. For a given statistical test performed at sensor-level, clusters were defined as spatially neighboring sensors exhibiting test statistics of the same sign (i.e., $t$-values for the analysis of neuromagnetic prediction correlates; Spearman $\rho$-values for the behavioral correlation analysis) or positive input parameters (i.e., $k^{\prime}$, vector norm, or vector angle for the SHI analyses) and $p$ < 0.05 (uncorrected). Adjacent sensors were defined based on the CTF275_neighb.mat template in Fieldtrip^53^.

For each resulting cluster, absolute values of the test statistics across all sensors within the current cluster were summed up to create a cluster summary statistic. Null distributions of cluster statistics were computed by randomly permuting the data independently for each subject, while permutation order was kept constant across sensors. Based on this permuted data, statistical assessment was again performed for each sensor, retaining the maximum cluster statistic across all clusters. This process was repeated 1000 times, yielding a null distribution of 1000 shuffled cluster statistics. $p$-values were assigned to clusters computed for experimental data relative to cluster statistics computed for the shuffled null distribution. For the analyses of final tone pitch prediction and behavioral correlation, clusters were considered significant if their cluster statistic lies in the top 2.5th percentile of shuffled data, corresponding to a two-tailed test at $p$ < 0.05. For the effects of $k^{\prime}$, vector norm, and vector angle, clusters were considered significant if their cluster statistic lies in the top (for $k^{\prime}$ and vector norm) or bottom (for vector angle) 5th percentile of shuffled data, corresponding to a one-tailed test at $p$ < 0.05. Measures of effect size for clusters in the original data (*d*_cluster_) were defined as the number of SDs by which the original cluster statistic exceeds the mean of the null distribution derived from permutated data.

### Reporting summary

Further information on research design is available in the Nature Research Reporting Summary linked to this article.

## Supplementary information

Supplementary Information

Reporting Summary

## Data Availability

Source data and code to reproduce all main text and supplementary figures are provided as supplementary information. Due to the large file size of raw MEG datasets, the raw dataset generated during the current study is available by request to the corresponding author. Source data are provided with this paper.

## Source data

Source data and code for figures Source data for Figure 2

## Code availability

We used publicly available open source software toolboxes and custom scripts written in MATLAB to analyze our data. Code supporting this study is available at a dedicated Github repository [https://github.com/BiyuHeLab/NatCommun_Baumgarten2021].
