# Supplementary material for: Neural integration underlying naturalistic prediction flexibly adapts to varying sensory input rate
Source: Nat Commun. 2021 May 11;12:2643. doi: 10.1038/s41467-021-22632-z (PMC8113607; doi:10.1038/s41467-021-22632-z)
Supplement: Supplementary file 1 — Supplementary Information [file 41467_2021_22632_MOESM1_ESM.pdf]

# Neural integration underlying naturalistic prediction flexibly adapts to varying sensory input rate

## Supplementary Information

**Baumgarten et al.**

## Supplementary Notes

### Effects of familiarity on prediction correlates

In order to investigate learning effects related to subjects' familiarity with the presented tone sequences, we split trials into early vs. late presentations. To this end, we determined all trials in which a unique tone sequence was presented for each subject. For the analysis of behavioral prediction effects, uniqueness was defined by a unique tone duration, $p^*_{34}$, $p_{34}$, and beta level. For the analysis of neural prediction effects, uniqueness was defined by a unique tone duration, $p^*_{34}$, and beta level (i.e., since the analysis of neural prediction effects focusses on neural activity during $p_{33}$, $p_{34}$ does not constitute trial uniqueness; Fig. 1B). For each unique tone sequence, we then separated the first half of presented trials from the last half of presented trials. Next, we recomputed the behavioral and neural prediction effects (all $n$ = 20) separately using the first or last half of presented trials. Otherwise, analyses were performed as described in the main manuscript.

Regarding the behavioral effects, significant main effects of $p_{34}$ collapsed across tone durations could be replicated for both the first ($F_{5,95}$ = 18.66, $p$ < 0.001, $\eta^2_p$ = 0.5) and the second half of the trials ($F_{2,41}$ = 10.46, $p$ < 0.001, $\eta^2_p$ = 0.36). Likewise, significant $p_{34} \times p^*_{34}$ interaction effects could be replicated for all tone duration conditions as well as collapsed across all tone durations for the first half of trials (150 ms: $F_{5,95}$ = 9.81, $p$ < 0.001, $\eta^2_p$ = 0.3; 300 ms: $F_{10,190}$ = 12.1, $p$ < 0.001, $\eta^2_p$ = 0.39; 600 ms: $F_{5,93}$ = 17.26, $p$ < 0.001, $\eta^2_p$ = 0.48; all tone durations: $F_{4,75}$ = 29.69, $p$ < 0.001, $\eta^2_p$ = 0.61). For the second half of trials, significant $p_{34} \times p^*_{34}$ interaction effects could be replicated for all tone duration conditions as well as collapsed across all tone durations (150 ms: $F_{10,190}$ = 7.29, $p$ < 0.001, $\eta^2_p$ = 0.28; 300 ms: $F_{4,83}$ = 12.95, $p$ < 0.001, $\eta^2_p$ = 0.41; 600 ms: $F_{4,80}$ = 15.65, $p$ < 0.001, $\eta^2_p$ = 0.45; all tone durations: $F_{3,60}$ = 23.5, $p$ < 0.001, $\eta^2_p$ = 0.55). This shows that subjects performed effective prediction of upcoming tone pitch in both the first and the second half of the experiment.

For the neural correlates of prediction, effects were also consistent across the first and second halves of the experiment (Supplementary Fig. 2). While the prediction effect reached significance following cluster-based permutation test in the first half (150 ms and 300 ms tone duration) and the second half (300 ms and 600 ms tone duration) of the experiment, the topography of the prediction effects is consistent across both halves of the experiment and all tone-duration conditions, and similar to the original results based on all trials. Compared with the original results (Fig. 3A & Supplementary Fig. 1), sensor clusters reached significance in fewer time windows due to a loss of power with only half of the trials analyzed in each time window.

Taken together, neither behavioral nor neural prediction effects seem to be driven mainly by an effect of learning (i.e., effects are more prominent in later trials) or an effect of fatigue (i.e., effects are more prominent in earlier trials). Instead, results remained comparatively similar between early and late blocks. We attribute this stability to the large set of individual tone sequences (9 sequences * 3 tone duration conditions), which is expected to hamper explicit recognition and learning of individual tone sequences.

## Supplementary Figures

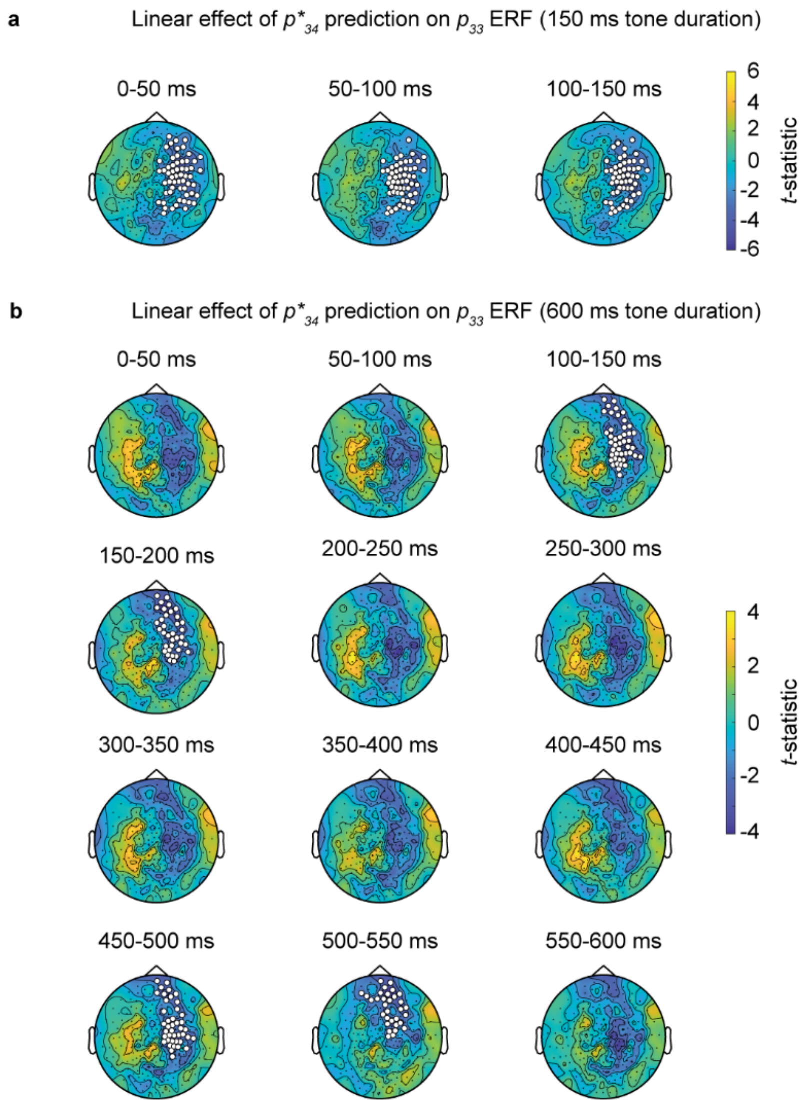


**Supplementary Fig. 1. Neuromagnetic correlates of prediction for 150 ms and 600 ms tone duration conditions (*n* = 20 participants).**

**a** Group-level neuromagnetic correlates of theoretically predicted final tone ($p_{34}^{*}$) prediction for the 150 ms tone duration condition. Non-baseline-corrected neuromagnetic activity averaged across 50 ms time windows during the penultimate tone ($p_{33}$) was regressed onto $p_{34}^{*}$ to reveal sensor clusters where neuromagnetic activity is predictive of future tone pitch. Topoplots show $t$-values corresponding to a group-level one-sample $t$-test on regression coefficients for each sensor and time window. White dots indicate significant predictive processing clusters (all $p <$ 0.05, cluster-based permutation test, two-tailed).

**b** Group-level neuromagnetic correlates of $p_{34}^{*}$ prediction for the 600 ms tone duration condition. Format is the same as a.

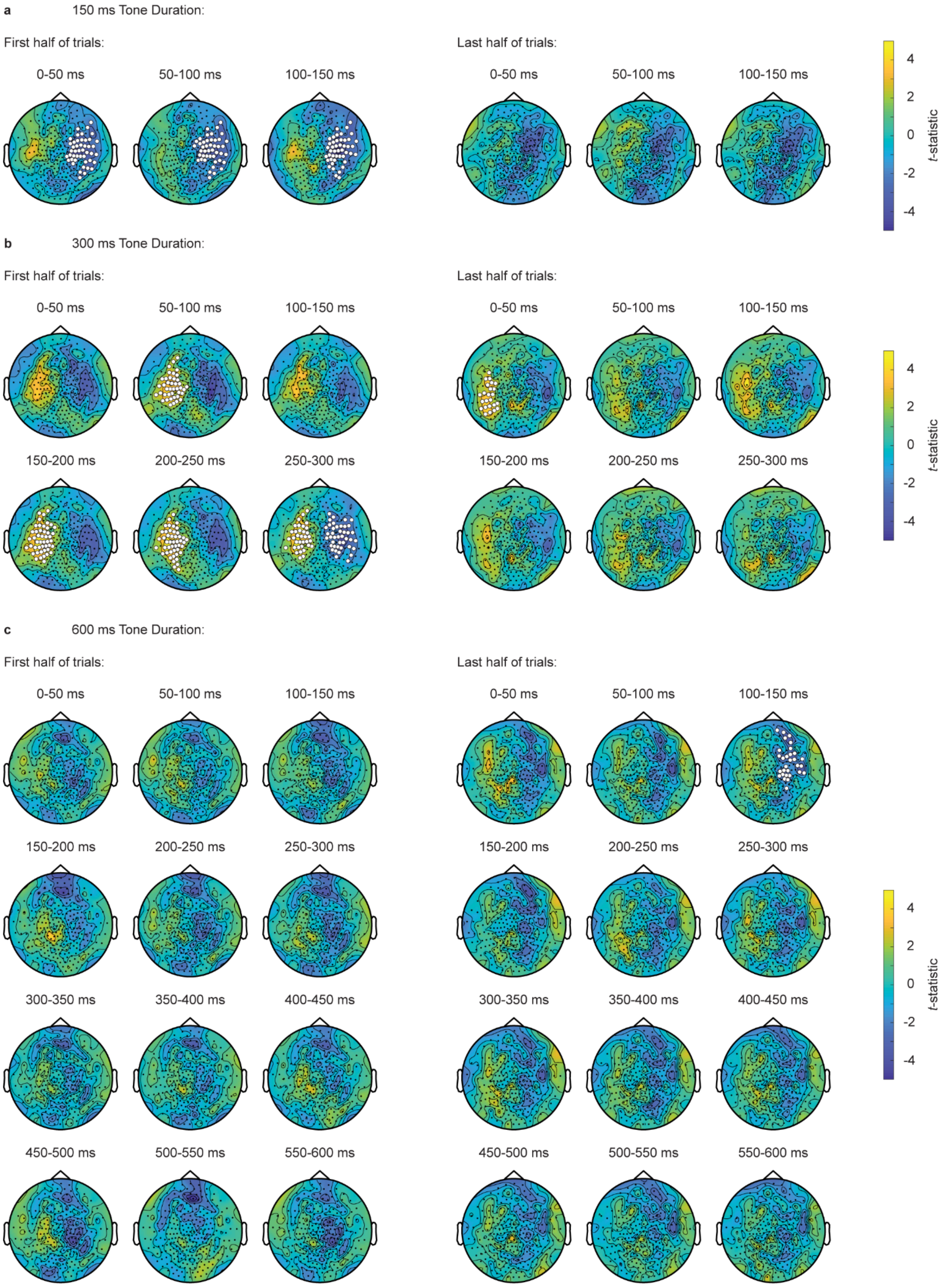
a
150 ms Tone Duration:
First half of trials:
0-50 ms
50-100 ms
100-150 ms
Last half of trials:
0-50 ms
50-100 ms
100-150 ms
4
2
0
-2
-4
t-statistic
b
300 ms Tone Duration:
First half of trials:
0-50 ms
50-100 ms
100-150 ms
150-200 ms
200-250 ms
250-300 ms
Last half of trials:
0-50 ms
50-100 ms
100-150 ms
150-200 ms
200-250 ms
250-300 ms
4
2
0
-2
-4
t-statistic
c
600 ms Tone Duration:
First half of trials:
0-50 ms
50-100 ms
100-150 ms
150-200 ms
200-250 ms
250-300 ms
300-350 ms
350-400 ms
400-450 ms
450-500 ms
500-550 ms
550-600 ms
Last half of trials:
0-50 ms
50-100 ms
100-150 ms
150-200 ms
200-250 ms
250-300 ms
300-350 ms
350-400 ms
400-450 ms
450-500 ms
500-550 ms
550-600 ms
4
2
0
-2
-4
t-statistic

**Supplementary Fig. 2: Effects of familiarity on neural correlates of prediction (*n* = 20 participants).**

**a** Group-level neuromagnetic correlates of prediction for the 150 ms tone duration condition, broken down by the first and second half of the experiment. Non-baseline-corrected neuromagnetic activity averaged across 50 ms time windows was regressed onto the theoretically predicted final tone pitch ($p_{34}^{*}$) to reveal sensor clusters where neuromagnetic activity is predictive of future tone pitch. Topoplots show $t$-values corresponding to a group-level one-sample $t$-test on regression coefficients for each sensor and time window. White dots indicate significant predictive processing clusters (all $p$ < 0.05, cluster-based permutation test, two-tailed).

**b** Group-level neuromagnetic correlates of prediction for the 300 ms tone duration condition, broken down by the first and second half of the experiment. Format is the same as a.

**c** Group-level neuromagnetic correlates of prediction for the 600 ms tone duration condition, broken down by the first and second half of the experiment. Format is the same as a.

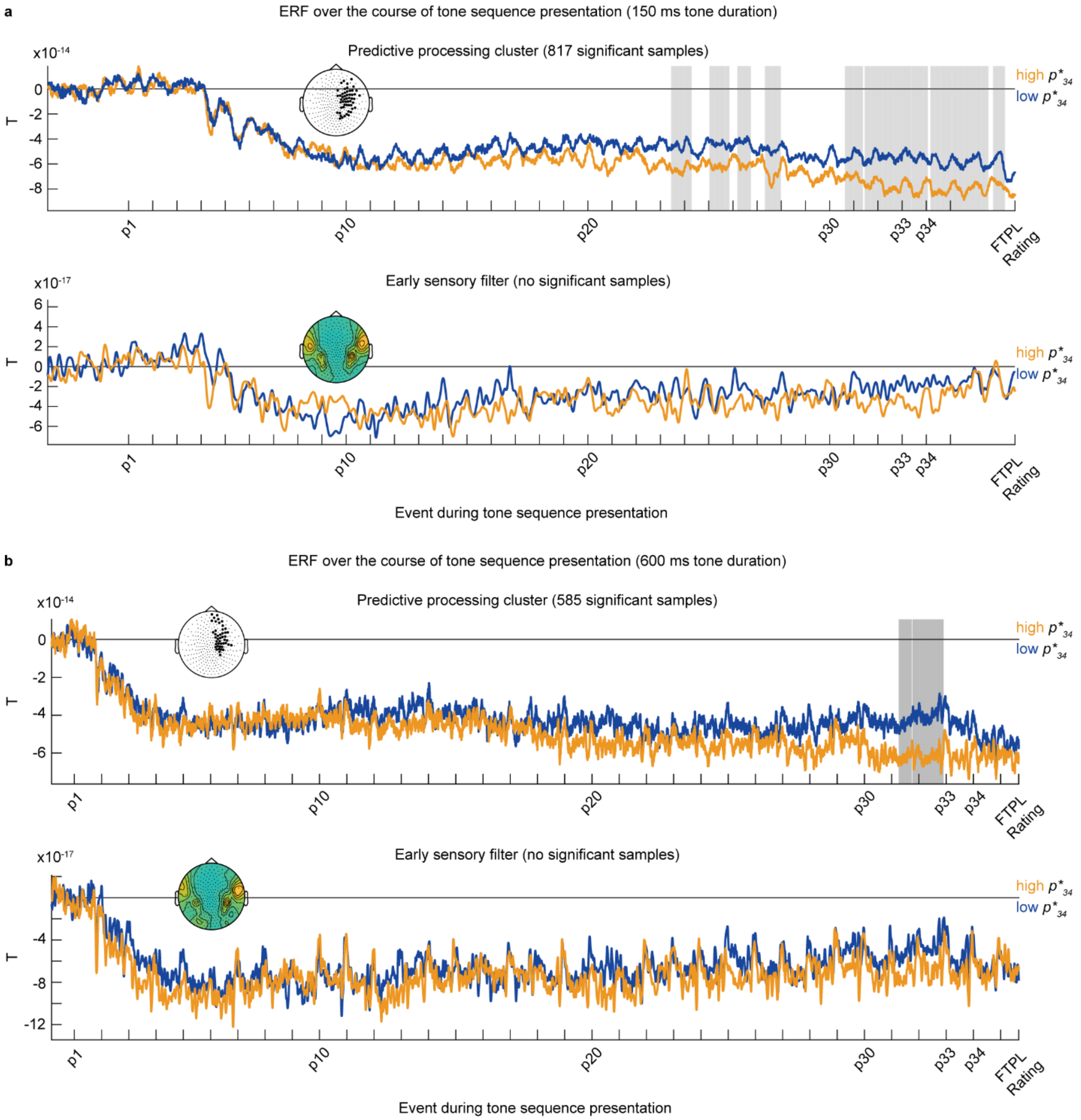


**Supplementary Fig. 3. Event-related fields (ERF) over the course of tone sequence presentation for the 150 ms and 600 ms tone duration conditions (*n* = 20 participants).**

**a** Neural activity time-locked to tone presentation over the course of the entire tone sequence ($p_1$ = tone 1 within the current sequence) for sequences with low vs. high theoretically predicted final tone ($p_{34}^*$), for the 150 ms tone duration condition. Time-locked activity is shown for a predictive processing cluster (top panel; defined for 100-150 ms time window; inset and Supplementary Fig. 1a) and early sensory filters (bottom panel; inset shows corresponding sensor weights). Gray shading indicates significant differences between trials with low vs. high $p_{34}^*$ (all $p$ < 0.05, cluster-based permutation test, two-tailed). Data are presented as mean across participants.

**b** Same as a, but for the 600 ms tone duration condition. The predictive processing cluster was defined from the 600 ms tone duration condition using the 100-150 ms time window (inset and Supplementary Fig. 1b).

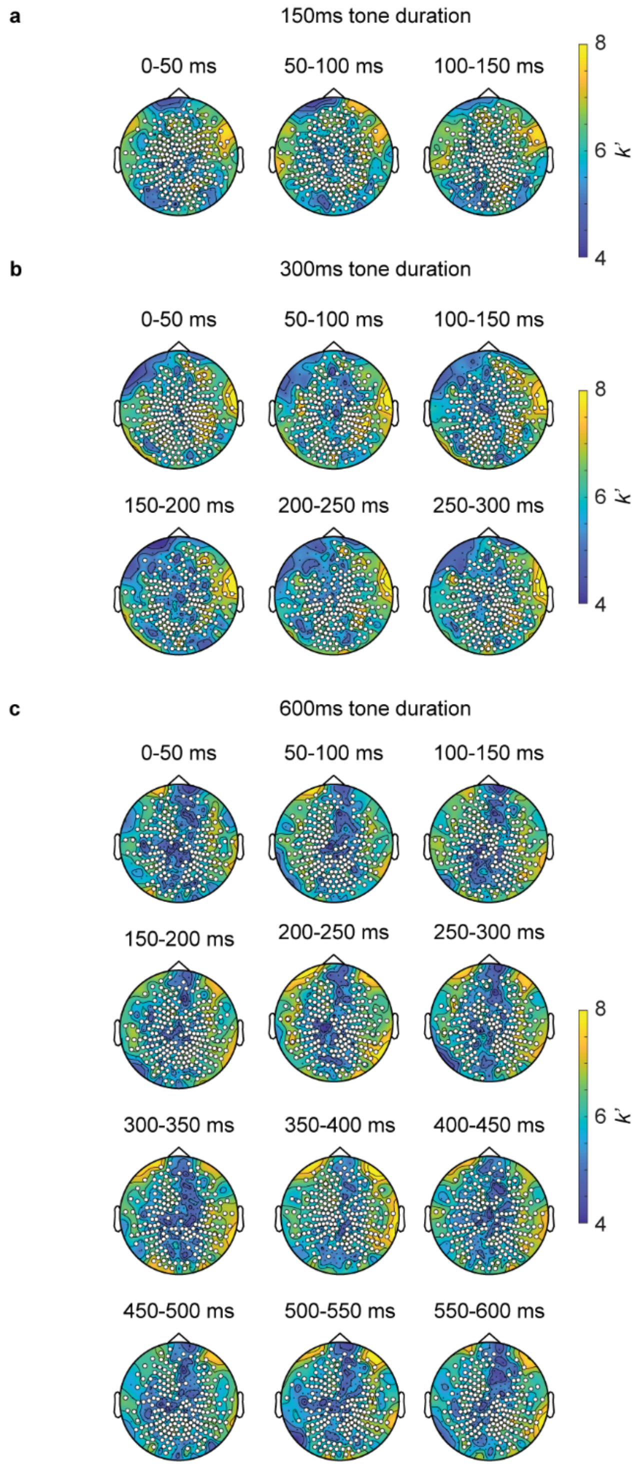


**Supplementary Fig. 4. Sensory history integration (SHI) effects per tone duration condition (*n* = 20 participants).**

**a** Group-level neuromagnetic correlates of SHI for the 150 ms tone duration condition. Topoplots show the number of preceding tones best explaining neuromagnetic activity ($k'$-values) for each sensor and time window indicating the number of preceding tones best explaining non-baseline-

corrected neuromagnetic activity (see Fig. 4 for analysis schematic). White dots indicate significant sensor clusters (all $p < 0.001$, cluster-based permutation test, one-tailed).

**b** Group-level neuromagnetic correlates of SHI for the 300 ms tone duration condition. Same format as a.

**c** Group-level neuromagnetic correlates of SHI for the 600 ms tone duration condition. Same format as a.

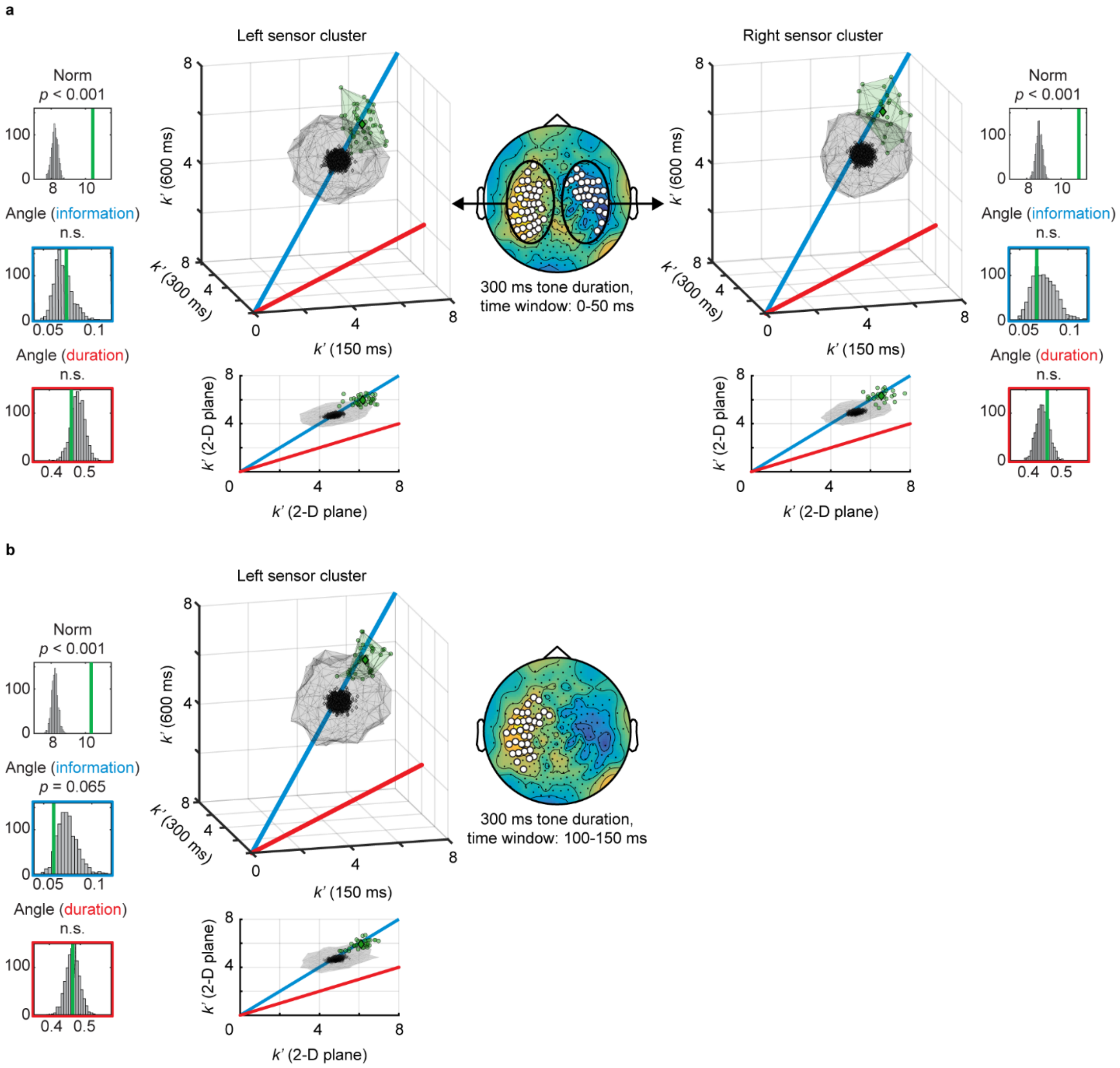


**Supplementary Fig. 5. Sensory history tracking analysis and comparison across tone duration conditions for predictive processing clusters (*n* = 20 participants).**

**a** Across-tone duration-condition comparison of the number of preceding tones best explaining neuromagnetic activity ($k'$-values) from the left and right predictive processing cluster (300 ms tone duration condition, 0-50 ms time window; Fig. 5a shows the 50-100 ms time window; see Figure 3a for all predictive processing clusters from the 300 ms tone duration condition). $k'$-values from experimental data in both sensor clusters (3-D and 2-D plots, green dots) reside significantly (left and right top histograms, $p < 0.001$, non-parametric permutation test, one-tailed) further away from origin at $k' \approx 6$ (i.e., 7 integrated tones) than shuffled data (gray dots).

**b** Same as a, but for predictive processing clusters defined in the 100-150 ms time window from the 300 ms tone duration condition data. $k'$-values from experimental data (3-D and 2-D plots, green dots) reside significantly (top histogram, $p < 0.001$, non-parametric permutation test, one-tailed) further away from origin at $k' \approx 6$ (i.e., 7 integrated tones) than shuffled data (gray dots).

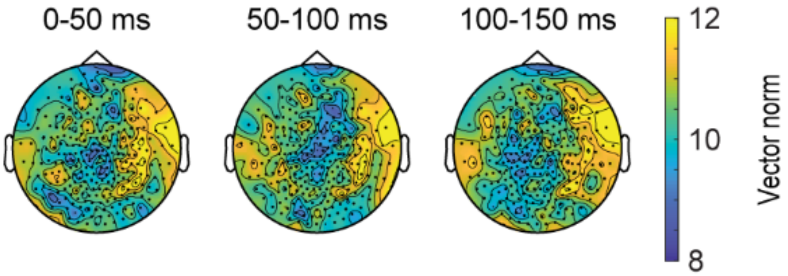


**Supplementary Fig. 6. Comparison of sensory history integration vector norm effects for the entire sensor array (*n* = 20 participants).**

Comparison of vector norm computed for the number of preceding tones best explaining neuromagnetic activity computed from experimental ($k'$-values) vs. shuffled data ($k'_{shuff}$-values) for the entire sensor array. Topoplots show vector norm values for each sensor and time windows from 0-150 ms.

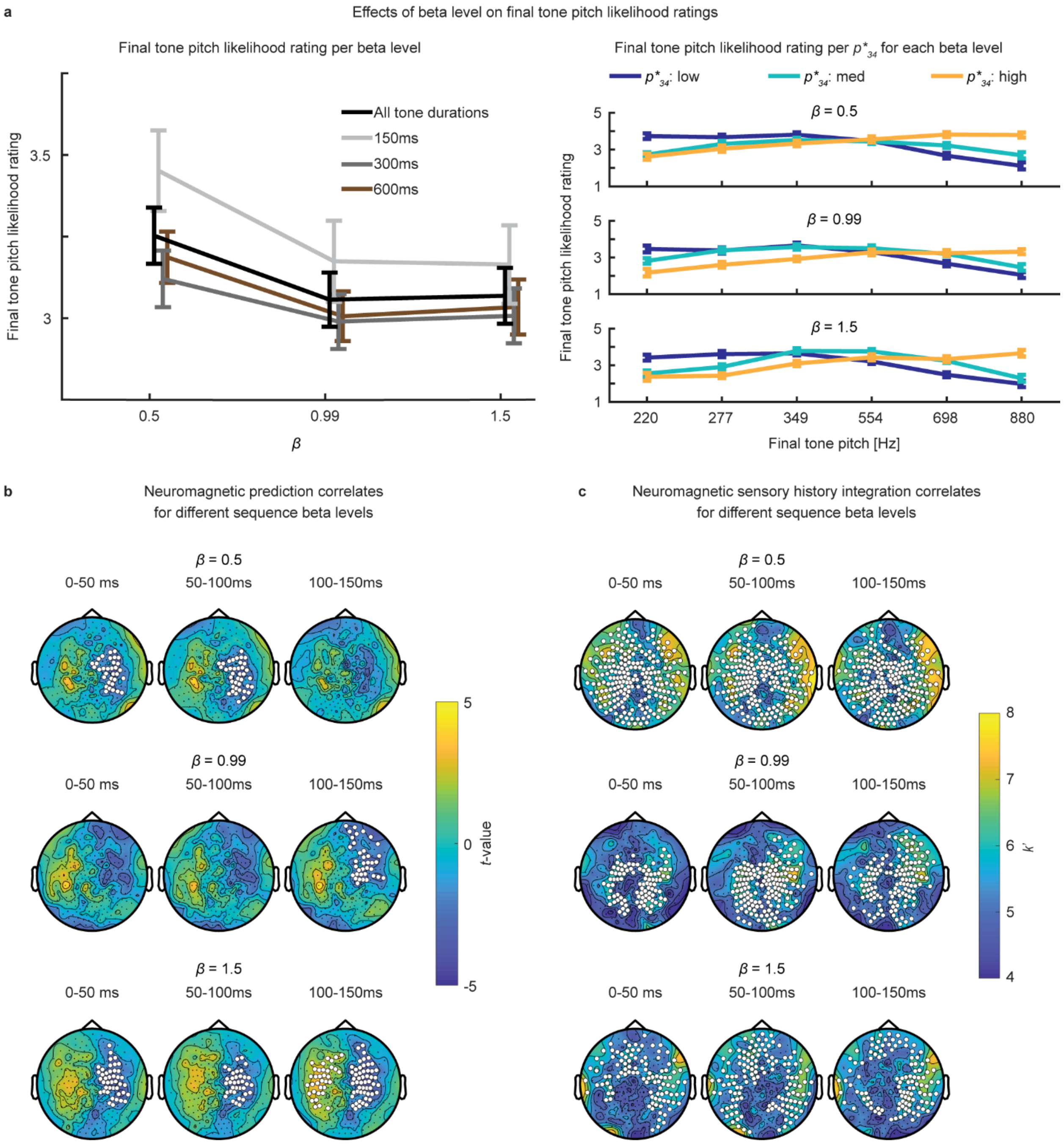


**Supplementary Fig. 7. Effects of sequence beta level (*n* = 20 participants).**

**a** Group-level final tone pitch likelihood ratings as a function of beta level. Left inset: Final tone pitch ratings (y-axis; 1 = unlikely, 5 = likely) per beta level as a function of tone duration condition and averaged across all tone durations. Right inset: Final tone pitch likelihood rating as a function of presented final tone pitch ($p_{34}$, x-axis) and theoretically predicted final tone pitch ($p_{34}^{*}$; color-coded) for each beta level. The $p_{34} \times p_{34}^{*}$ interaction effect is significant for each beta level (Factors: $p_{34}^{*}$, $p_{34}$, tone duration; beta level 0.5: $F_{5,92} = 21.64$, $p < 0.001$, $\eta_p^2 = 0.53$; beta level 0.99: $F_{5,91} = 15.9$, $p < 0.001$, $\eta_p^2 = 0.46$; beta level 1.5: $F_{4,71} = 23.55$, $p < 0.001$, PE $\eta_p^2 = 0.55$). Final

tone pitch ratings are averaged across all tone duration conditions. Data are presented as mean ± SEM across participants.

**b** Neuromagnetic prediction correlates (linear effect of $p_{34}^{*}$ prediction on the ERF during the penultimate tone ($p_{33}$)) for tone sequences with different beta levels. Upper row: Neuromagnetic prediction correlates for tone sequences with beta level 0.5. Topoplots show $t$-values corresponding to a group-level one-sample $t$-test on regression coefficients for each sensor and time window. White dots indicate significant predictive processing clusters (all $p$ < 0.05, cluster-based permutation test, two-tailed). Middle row: Neuromagnetic prediction correlates for tone sequences with beta level 0.99. Lower row: Neuromagnetic prediction correlates for tone sequences with beta level 1.5.

**c** Sensory history integration (SHI) effects for tone sequences with different beta levels. Upper row: SHI effects for tone sequences with beta level 0.5. Topoplots show the number of preceding tones best explaining neuromagnetic activity ($k'$-values) for each sensor and time window. White dots indicate significant sensor clusters (all $p$ < 0.001, cluster-based permutation test, two-tailed). Middle row: Sensory history integration effects for tone sequences with beta level 0.99. Lower row: Sensory history integration effects for tone sequences with beta level 1.5.

**Supplementary Table 1. Comparison of vector norm and vector angle per time window (*n* = 20 participants).**

| Results for data projected into three-dimensional space (non-parametric permutation test, one-tailed). | | | |
|---|---|---|---|
| Time window and predictive processing sensor cluster (computed for the 300 ms tone duration condition) | Vector norm | Vector angle to information line [radians] | Vector angle to duration line [radians] |
| 0-50 ms – left sensor cluster | 10.42; $p < 0.001$ | 0.07; $p = 0.7$ | 0.47; $p = 0.2$ |
| 0-50 ms – right sensor cluster | 11.05; $p < 0.001$ | 0.07; $p = 0.3$ | 0.47; $p = 0.7$ |
| 50-100 ms – left sensor cluster | 10.67; $p < 0.001$ | 0.06; $p = 0.014$ | 0.51; $p = 0.8$ |
| 50-100 ms – right sensor cluster | 10.4; $p < 0.001$ | 0.08; $p = 0.7$ | 0.45; $p = 0.2$ |
| 100-150 ms – left sensor cluster | 10.33; $p < 0.001$ | 0.06; $p = 0.065$ | 0.48; $p = 0.5$ |
| Results for data projected into two-dimensional space (non-parametric permutation test, one-tailed). | | | |
| Time window and predictive processing sensor cluster (computed for the 300 ms tone duration condition) | Vector norm | Vector angle to information line [radians] | Vector angle to duration line [radians] |
| 0-50 ms – left sensor cluster | 10.41; $p < 0.001$ | 0.05; $p = 0.7$ | 0.47; $p = 0.2$ |
| 0-50 ms – right sensor cluster | 11.03; $p < 0.001$ | 0.05; $p = 0.4$ | 0.47; $p = 0.7$ |
| 50-100 ms – left sensor cluster | 10.66; $p < 0.001$ | 0.03; $p = 0.034$ | 0.51; $p = 0.8$ |
| 50-100 ms – right sensor cluster | 10.38; $p < 0.001$ | 0.06; $p = 0.8$ | 0.45; $p = 0.2$ |
| 100-150 ms – left sensor cluster | 10.32; $p < 0.001$ | 0.03; $p = 0.057$ | 0.47; $p = 0.5$ |
