# Supplementary material for: Neural integration underlying naturalistic prediction flexibly adapts to varying sensory input rate
Source: Nat Commun. 2021 May 11;12:2643. doi: 10.1038/s41467-021-22632-z (PMC8113607; doi:10.1038/s41467-021-22632-z)
Supplement: Supplementary file 2 — Reporting Summary [file 41467_2021_22632_MOESM2_ESM.pdf]

nature research

Corresponding author(s): Biyu J He

Last updated by author(s): Feb 25, 2021

Reporting Summary

Nature Research wishes to improve the reproducibility of the work that we publish. This form provides structure for consistency and transparency in reporting. For further information on Nature Research policies, see our Editorial Policies and the Editorial Policy Checklist.

Statistics

For all statistical analyses, confirm that the following items are present in the figure legend, table legend, main text, or Methods section.

n/a	Confirmed	
[ ]	[x]	The exact sample size (n) for each experimental group/condition, given as a discrete number and unit of measurement
[ ]	[x]	A statement on whether measurements were taken from distinct samples or whether the same sample was measured repeatedly
[ ]	[x]	The statistical test(s) used AND whether they are one- or two-sided<br>*Only common tests should be described solely by name; describe more complex techniques in the Methods section.*
[ ]	[x]	A description of all covariates tested
[ ]	[x]	A description of any assumptions or corrections, such as tests of normality and adjustment for multiple comparisons
[ ]	[x]	A full description of the statistical parameters including central tendency (e.g. means) or other basic estimates (e.g. regression coefficient) AND variation (e.g. standard deviation) or associated estimates of uncertainty (e.g. confidence intervals)
[ ]	[x]	For null hypothesis testing, the test statistic (e.g. F, t, r) with confidence intervals, effect sizes, degrees of freedom and P value noted<br>*Give P values as exact values whenever suitable.*
[x]	[ ]	For Bayesian analysis, information on the choice of priors and Markov chain Monte Carlo settings
[x]	[ ]	For hierarchical and complex designs, identification of the appropriate level for tests and full reporting of outcomes
[ ]	[x]	Estimates of effect sizes (e.g. Cohen's d, Pearson's r), indicating how they were calculated

Our web collection on statistics for biologists contains articles on many of the points above.

Software and code

Policy information about availability of computer code

Data collection	Data collection was performed with MATLAB (version 2017a). Experiment presentation was performed with the Psychophysics Toolbox (version 3).
Data analysis	Data analysis was performed with MATLAB (version 2017a), the Fieldtrip toolbox (version 20180725), and custom written codes. Code supporting this study is available at a dedicated Github repository [https://github.com/BiyuHeLab/NatCommun_Baumgarten2021].

For manuscripts utilizing custom algorithms or software that are central to the research but not yet described in published literature, software must be made available to editors and reviewers. We strongly encourage code deposition in a community repository (e.g. GitHub). See the Nature Research guidelines for submitting code & software for further information.

Data

Policy information about availability of data

All manuscripts must include a data availability statement. This statement should provide the following information, where applicable:

- Accession codes, unique identifiers, or web links for publicly available datasets
- A list of figures that have associated raw data
- A description of any restrictions on data availability

Due to the extensive file size of raw MEG datasets, the datasets generated and analyzed during the current study are available by request to the corresponding author. Source data files for all figures are provided as supplementary information.

Field-specific reporting

Please select the one below that is the best fit for your research. If you are not sure, read the appropriate sections before making your selection.

- [ ] Life sciences
- [x] Behavioural & social sciences
- [ ] Ecological, evolutionary & environmental sciences

For a reference copy of the document with all sections, see nature.com/documents/nr-reporting-summary-flat.pdf

Behavioural & social sciences study design

All studies must disclose on these points even when the disclosure is negative.

Study description	Study including quantitative recordings of behavior and neuromagnetic (MEG) data.
Research sample	The research sample consisted of healthy human volunteers (26 subjects in total; final sample: 20 subjects; 11 females; mean age 25.0 (19-34) y), including students and non-students. Sample is representative due to an age range covering young adults and balanced male-female ratio. Rationale for subject selection included availability and sufficiently high level of task performance.
Sampling strategy	A convenience sample was recruited through a volunteer subject pool at the National Institutes of Health, MD, USA. The sample size was based on comparable published MEG studies on neural prediction effects, as for instance in:<br><br>Maniscalco, B., Lee, J. L., Abry, P., Lin, A., Holroyd, T., & He, B. J. (2018). Neural integration of stimulus history underlies prediction for naturalistically evolving sequences. Journal of Neuroscience, 38(6), 1541-1557.
Data collection	Behavioral data was collected via computer (MatLab, Psychophysics Toolbox). Neuromagnetic data was recorded via a 275-channel CTF MEG scanner (VSM MedTech, Coquitlam, BC, Canada). Experimenters (B.M., J.L.L., M.W.F.) were present during data collection and monitored MEG data acquisition. Blinding was not used since the all subjects were presented with the same stimuli (within-subject paradigm).
Timing	Data collection was performed from July 2016 - May 2017.
Data exclusions	From the initial 26 subjects, six subjects were excluded due to either poor performance (i.e., not using the full range of the rating scale) or excessive MEG artifacts, yielding a final group of 20 subjects. Exclusion criteria were established prior to the beginning of the study.
Non-participation	No participants dropped out during data collection.
Randomization	Subjects were not allocated to experimental groups, since all subjects were presented with the same stimuli (within-subject paradigm). Stimuli were presented in randomized order.

Reporting for specific materials, systems and methods

We require information from authors about some types of materials, experimental systems and methods used in many studies. Here, indicate whether each material, system or method listed is relevant to your study. If you are not sure if a list item applies to your research, read the appropriate section before selecting a response.

Materials & experimental systems

n/a	Involved in the study	
x		Antibodies
x		Eukaryotic cell lines
x		Palaeontology and archaeology
x		Animals and other organisms
	x	Human research participants
x		Clinical data
x		Dual use research of concern

Methods

n/a	Involved in the study	
x		ChIP-seq
x		Flow cytometry
x		MRI-based neuroimaging

Human research participants

Policy information about studies involving human research participants

Population characteristics	See above.
Recruitment	Participants were recruited via flyers and e-mail. Since this is a common way of subject recruitment for human neuroscience subjects, we do not expect any resulting potential self-selection bias.
Ethics oversight	The study was approved by the Institutional Review Board of the National Institute of Neurological Disorders and Stroke (protocol #14-N-0002). All subjects provided written, informed consent.

Note that full information on the approval of the study protocol must also be provided in the manuscript.
